# Exposure to elevated glucocorticoid during development primes altered transcriptional responses to acute stress in adulthood

**DOI:** 10.1016/j.isci.2024.110160

**Published:** 2024-05-31

**Authors:** Min-Kyeung Choi, Alexander Cook, Kanak Mungikar, Helen Eachus, Anna Tochwin, Matthias Linke, Susanne Gerber, Soojin Ryu

**Affiliations:** 1 Living Systems Institute & Department of Clinical and Biomedical Sciences, University of Exeter, Stocker Road, EX4 4QD Exeter, UK; 2 Institute of Human Genetics, University Medical Center, Johannes Gutenberg University Mainz, Langenbeckstraße 1, 55131 Mainz, Germany

**Keywords:** Neuroscience, Behavioral neuroscience, Molecular neuroscience, Omics, Transcriptomics

## Abstract

Early life stress (ELS) is a major risk factor for developing psychiatric disorders, with glucocorticoids (GCs) implicated in mediating its effects in shaping adult phenotypes. In this process, exposure to high levels of developmental GC (hdGC) is thought to induce molecular changes that prime differential adult responses. However, identities of molecules targeted by hdGC exposure are not completely known. Here, we describe lifelong molecular consequences of hdGC exposure using a newly developed zebrafish double-hit stress model, which shows altered behaviors and stress hypersensitivity in adulthood. We identify a set of primed genes displaying altered expression only upon acute stress in hdGC-exposed adult fish brains. Interestingly, this gene set is enriched in risk factors for psychiatric disorders in humans. Lastly, we identify altered epigenetic regulatory elements following hdGC exposure. Thus, our study provides comprehensive datasets delineating potential molecular targets mediating the impact of hdGC exposure on adult responses.

## Highlights

• High level of developmental GC exposure (hdGC) alters adult behavior in zebrafish
• hdGC primes exaggerated endocrine and transcriptional stress response in adults
• We identify a set of hdGC-primed genes in the brain and their associated functions
• We identify lasting impact of hdGC exposure on epigenetic modulator expressions

## Graphical abstract

**Figure:**
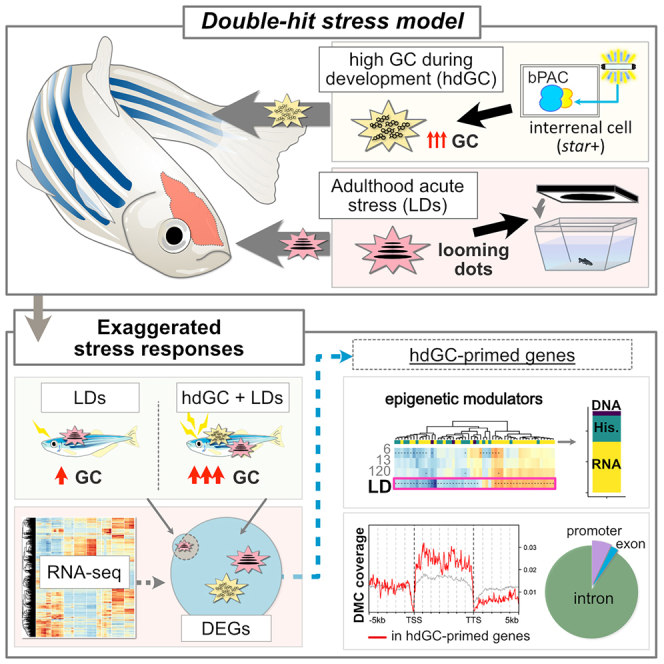

## Introduction

Early life stress (ELS) affects healthy development and aging as well as susceptibility to psychiatric disorders in humans.^1^^,^^2^ Studies in humans and animals suggest ELS may increase the risk of psychiatric diseases by affecting sensitivity to future stress exposure.^3^^,^^4^^,^^5^^,^^6^ However, the mechanism by which ELS sensitizes responses to future stress responses in adulthood remains poorly understood. In vertebrates, a stress response is mediated by a highly conserved neuroendocrine system called the Hypothalamic-Pituitary-Adrenal axis (HPA-axis) in humans^7^ and the Hypothalamic-Pituitary-Interrenal axis (HPI-axis) in fish whose activation result in the release of glucocorticoids (GCs).^8^ GCs have pleiotropic effects on many aspects of animal physiology and exposure to elevated GCs during development can cause long-term alteration of behavior, physiology, and stress regulation.^2^^,^^9^^,^^10^ However, how exposure to high levels of GC during development predisposes individuals to adulthood dysfunction is poorly understood. One potential mechanism is long-lasting epigenetic changes, which can modify how animals respond to stress in adulthood. For example, altered DNA methylation at the glucocorticoid receptor (GR) gene promoter induced by poor maternal care is associated with adulthood alterations in histone acetylation, DNA methylation, transcription factor binding, GR expression, and the HPA response to stress.^11^ Moreover, a recent study identified differentially expressed genes (DEGs) “primed” by ELS in specific regions of ELS-exposed mouse brains.^12^ These genes showed enhanced differential expression in ELS-exposed animals in adulthood upon re-exposure to stress and may be associated with behavioral changes in adulthood. Another study identified direct GC-primed DEGs and associated long-lasting DNA methylation alterations using a GC (dexamethasone)-exposed human hippocampal progenitor cell line.^13^ These studies show that ELS or developmental GC exposure results in distinct responses to stress later in life by priming and maintaining long-term alteration of specific genes. However, so far, ELS- or GC-primed genes have only been identified in specific regions of the brain or cell lines, and brain-wide GC-primed molecular alterations have not been investigated.

Here we report whole-brain transcriptomic alterations of high GC-exposed fish across the life course and upon acute stress exposure in adulthood. Interestingly, when subjected to acute stress in adulthood, hdGC-exposed fish show highly exaggerated endocrine and transcriptional responses, indicating that adult stress is processed differently depending on the GC exposure history of an animal. The set of genes primed by hdGC exposure was enriched in gene sets associated with human neuropsychiatric disorders, suggesting the similarity of molecular mechanisms by which early GC exposure leads to altered adult functions among vertebrates. We identify hitherto uncharacterized novel hdGC-primed genes that are enriched in synapse and neuronal signaling function. Whole-brain DNA methylation analysis identified some of them as direct targets of hdGC-mediated epigenetic modifications. Lastly, the expression of many epigenetic modulators affecting RNA processing, histone modification, and DNA modification is altered following acute stress in hdGC-exposed individuals, which can contribute to the different physiological and behavioral responses following adult stress exposure in later stages of life.

## Results

### Double-hit zebrafish stress model exhibits altered adulthood stress response

**Figure 1:**
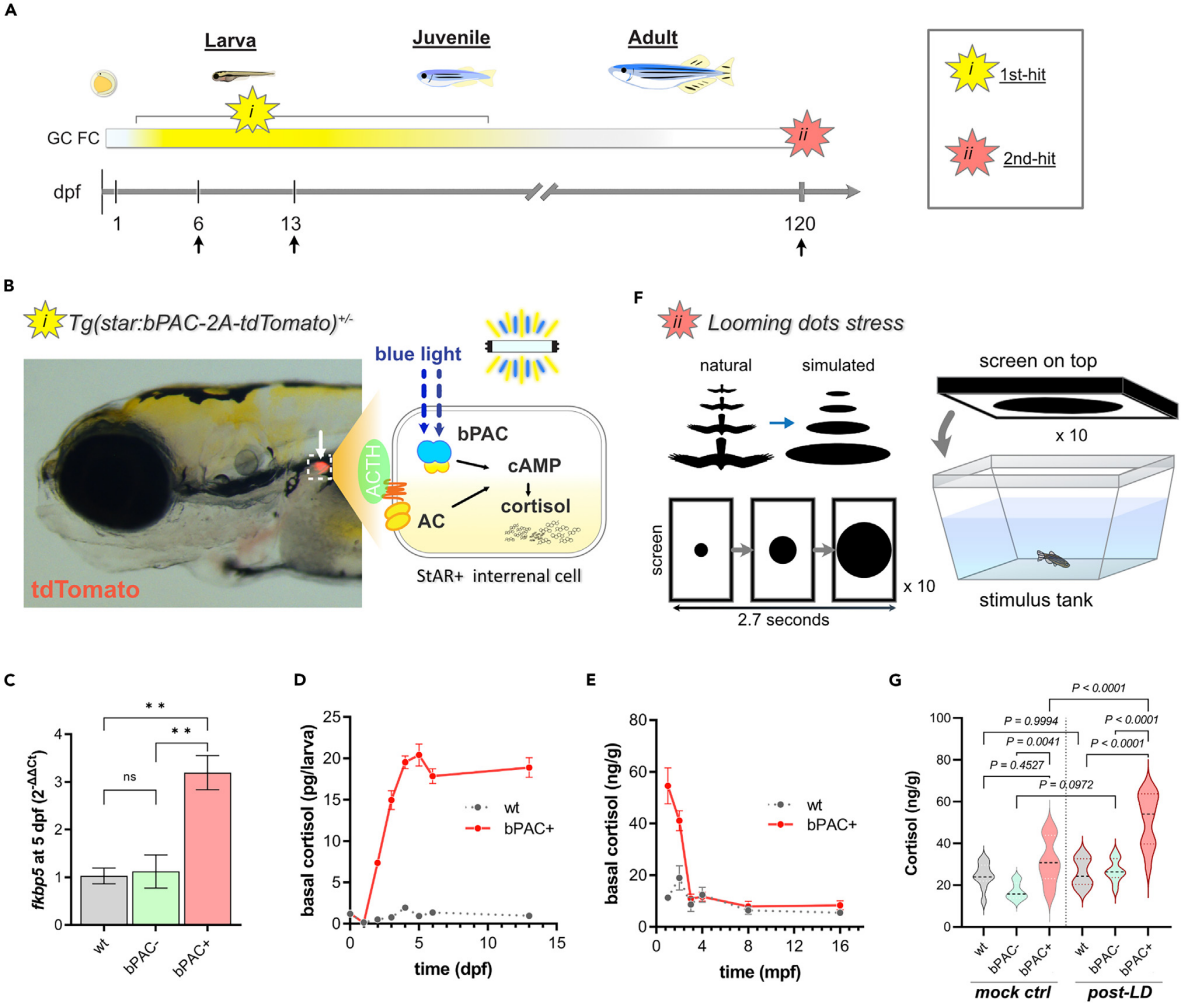
Double-hit stress model shows an elevation of cortisol during development and upon acute stress exposure in adulthood (A) A scheme illustrating the strategy underlying the double-hit zebrafish stress model which combines *i)* optogenetic cortisol elevation during development and *ii)* adulthood acute stress exposure. Arrows indicate sample collection times points for RNA-seq. (B) An image of *Tg(star:bPAC-2A-tdTomato)*^*ex300*^ larva at 5 dpf. The transgene is expressed solely in the interrenal gland (white arrow). A schematic drawing representing the intracellular events within the steroidogenic interrenal cells of the transgenic fish (dotted box). Endogenous GC production is stimulated by ACTH signaling. bPAC mimics ACTH signaling by increasing cAMP when exposed to blue light. (C) One of the well-known GR signaling markers, *fkbp5*, was significantly upregulated in bPAC+ larvae compared to bPAC- and wild-type control. ∗∗ = *p* < 0.01. Tukey’s multiple comparisons test following one-way ANOVA, F (2, 6) = 16.25, *p* = 0.0038. (D and E) Basal cortisol level at larval, juvenile, and adult stages (for animal numbers used in this experiment, see the STAR Methods). (F) A schematic drawing of the acute stress paradigm. Looming dots mimic approaching predators from above (Cook et al. 2023). (G) bPAC+ showed significantly higher cortisol levels following LD, compared to handling controls (no LD presented on the screen). *n* = 6 per group, Tukey’s multiple comparisons following one-way ANOVA; F(5, 54) = 19.31, *p* < 0.0001. Abbreviations: dpf: day post fertilization, mpf: month post fertilization, GC: glucocorticoid, ACTH: Adrenocorticotropic Hormone, AC: adenylyl cyclase, cAMP: Cyclic adenosine monophosphate, FC: fold change, LD: Looming dots, wt: wild type, ctrl: control, bPAC: *beggiatoa* photoactivated adenylyl cyclase. Error bars represent mean ± SEM.

We developed a double-hit stress model using zebrafish which combines exposure to a high level of GC during development with acute adult stress exposure (Figure 1A). First, we used our previously reported optogenetic transgenic model *Tg(star:bPAC-2A-tdTomato)*^*uex300*^ where the elevation of endogenous cortisol (GC in fish) is induced by blue light which activates *beggiatoa* photoactivated adenylyl cyclase (bPAC), expressed specifically in steroidogenic interrenal cells^14^^,^^15^^,^^16^ (Figure 1B). The fish interrenal gland, a homolog of the adrenal gland in mamals, is composed of two cell types: aminergic chromaffin cells and steroidogenic interrenal cells, the latter of which produce glucocorticoids (GCs). We established that *Tg(star:bPAC-2A-tdTomato)*^*uex300*^ raised under ambient light containing blue light (hitherto referred to as bPAC+) showed increased cortisol levels and expression of *fkbp5*, one of the known GR signaling marker genes,^17^^,^^18^^,^^19^ at larval stages^15^^,^^16^ (Figure 1C). Elevated cortisol levels through larval and juvenile stages were detected in bPAC+, but not maintained until adulthood (after 3 months) allowing us to achieve a robust and persistent increase in endogenous cortisol levels during development (Figures 1D and 1E). Throughout the study, bPAC+ fish were compared to bPAC-, siblings of bPAC+ which do not carry the transgene themselves but are offspring of a bPAC+ parent crossed to wild type. We also compared their phenotypes to wild-type TU, which is the same strain as bPAC+ and bPAC-, to determine possible effects resulting from elevated GC in parental generation (Figure S1). Secondly, for acute stress delivery, we utilized a looming dot stimulus (LD), which mimics an approaching predator^20^ (Figure 1F). bPAC+ adults exhibited an exaggerated endocrine response after LD exposure compared to wild-type or bPAC- adults and even an elevated GC level induced by handling before the LD exposure (Figure 1G), indicative of a highly sensitized endocrine response to acute stress in bPAC+.

### High glucocorticoid-exposed animals during development show altered behaviors in adulthood

**Figure 2:**
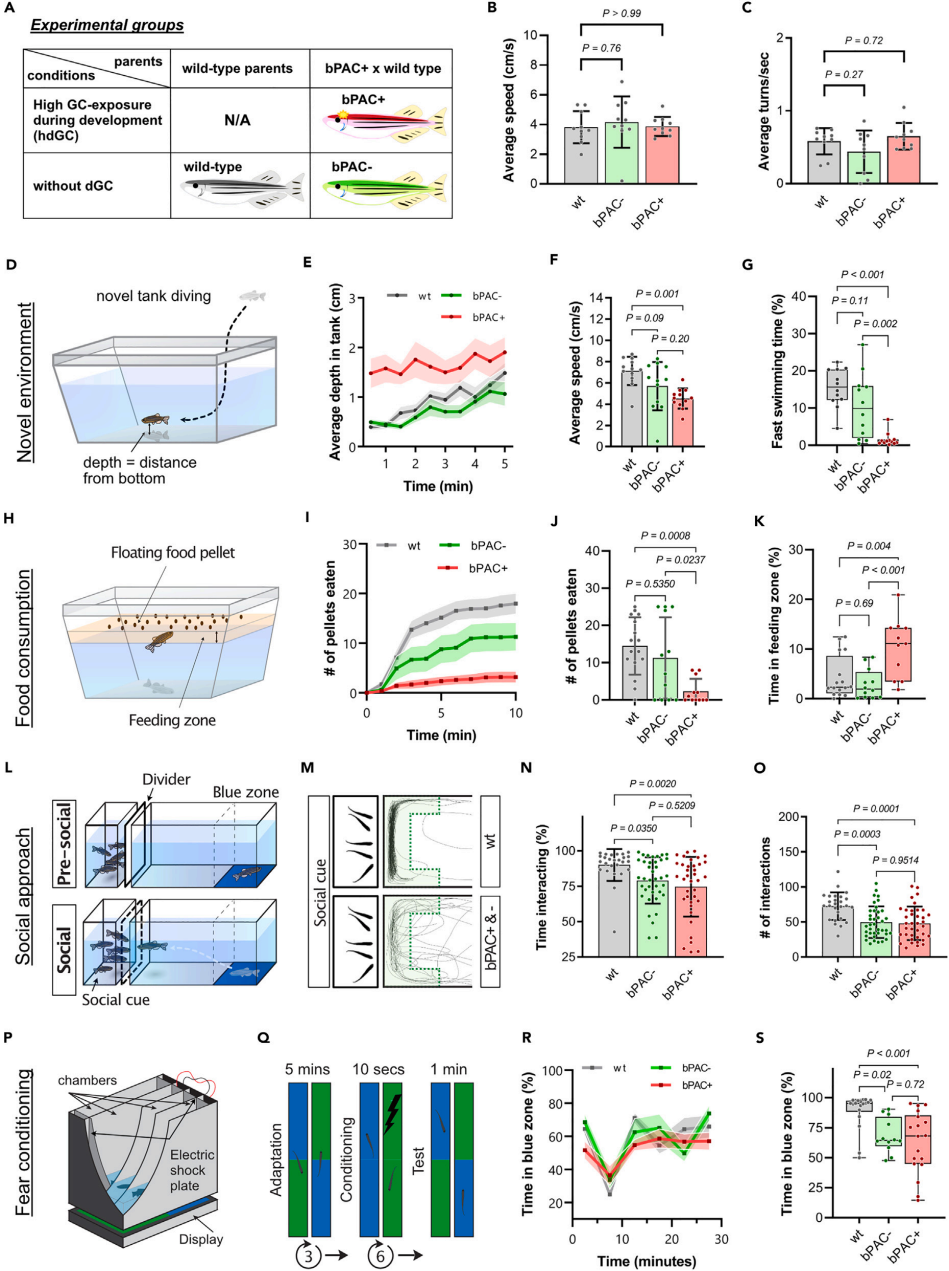
Altered behaviors in adult bPAC+ and bPAC- fish (A) A table depicting experimental groups. (B and C) No significant differences were observed in average speed nor turn frequency among wild type (wt), bPAC+, and bPAC-. Dunnett’s multiple comparisons followed one-way ANOVA, *n* = 10 per group. B) F(2, 27) = 0.2248, *p=0.80*; C) F(2, 27) = 2.332, *p=0.12*. (D) A schematic drawing for the novel tank test. (E) Average depth as measured by average distance from the bottom of the tank was greater for wt and bPAC- compared to bPAC+. (F and G) bPAC+ fish showed a slower average speed than wild type and reduced fast swimming bouts compared to bPAC- and wt, *n* = 12 per group. (H) A schematic drawing for the feeding behavior test. (I and J) bPAC+ consumed fewer number of pellets within 10 min compared to wt or bPAC-, *n* = 16 for wt, *n* = 12 for bPAC-, *n* = 12 for bPAC+. (K) bPAC+ spent more time near the food compared to wt or bPAC-, *n* = 16 for wt, *n* = 12 for bPAC-, *n* = 11 for bPAC+. (L) Schematic figures for the social behavior test. (M) Representative traces of wt, bPAC+, and bPAC- when conspecifics are visible. The green shaded area indicates the interaction zone where wildtype fish typically spend most of their time when conspecifics are visible. (N and O) During this social phase, bPAC+ and bPAC- showed a significantly reduced duration and frequency of social interactions compared to the wt (Cook et al., 2023), *n* = 26 for wt, *n* = 38 for bPAC-, *n* = 37 for bPAC+. (P) A schematic drawing for a chamber used for the fear conditioning test. (Q) An electric shock was given to subject fish when they swam in the green-colored area. After conditioning, time spent in each color zone was measured. (R) No significant difference was observed in color preference prior to fear conditioning. (S) bPAC+ showed reduced preference for the safe zone color (blue) after the conditioning using an electric shock, *n* = 18 for wt, n- = 18 for bPAC+, *n* = 12 for bPAC-. Error bars on bar graphs indicate the mean ± SD while shading in line graphs represents the mean ± SEM. Tukey’s multiple comparisons followed one-way ANOVA. F) F(2, 33) = 7.806, *p=0.002*; G) F(2, 33) = 17.31, *p < 0.0001*; J) F(2, 37) = 8.364, *p=0.001*; K) F(2, 36) = 9.120, *p < 0.001*, N) F(2, 98) = 6.273, *p=0.0027*; O) F(2, 98) = 10.93, *p < 0.0001*; S) F(2, 45) = 8.927, *p < 0.001*.

To determine whether exposure to a high level of endogenous GC during development alters adult functions, we tested adult behavior encompassing different behavioral domains using wild type, bPAC-, and bPAC+ (Figure 2). We first established that they did not show significant differences in basal locomotor activity (Figures 2B and 2C). We then used a novel tank test to assess their adaptive responses in a novel environment^21^^,^^22^ (Figures 2D–2G). When placed in a novel tank, zebrafish typically first dive to the bottom whilst performing bouts of erratic high-speed swimming.^22^ There was no significant difference between bPAC- and wild types in average depth nor fast swimming time (Figures 2E and 2G). In contrast, bPAC+ fish showed reduced depth-preference (Figure 2E) and reduced fast-swimming compared to bPAC- and wild-type fish (Figure 2G). In addition, bPAC+ showed reduced average speed compared to wild type (Figure 2F). Next, we tested feeding behavior by counting the number of floating food pellets eaten by a fish during a 10-min interval (Figure 2H). Wild-type and bPAC- adult fish consumed on average 14.5 and 11.25 pellets, respectively while bPAC+ fish consumed fewer pellets (on average 2.33 pellets out of 25) (Figures 2I and 2J). This was not due to the lack of recognition nor interest in the food, as bPAC+ fish approached the food pellets and spent a longer time near the pellets (feeding zone) than bPAC- and wild-type fish (Figure 2K). Further, we analyzed social behavior using a new test that we recently established, which allows measuring both social approach and social interaction within the same paradigm (Figure 2L). While bPAC+ exhibited reduced social interactions compared to wild type, no significant difference was observed in bPAC+ compared to bPAC- (Figures 2M–2O). Similarly, whilst bPAC+ exhibited altered fear conditioning compared to wild type, there was no significant difference observed between bPAC+ and bPAC- in an associative learning test using a Pavlovian avoidance learning test^23^^,^^24^ (Figures 2P–2S). Thus, exposure to a high level of endogenous GC during development led to an exaggerated endocrine response upon acute stress exposure in adulthood and alteration in responses in a novel environment and feeding behaviors. For social and fear conditioning behaviors, exposure to high levels of GC during parental generation appears to be sufficient to modify adult behavior.

### Distinct transcriptional alterations in high glucocorticoid-exposed fish across the life course

**Figure 3:**
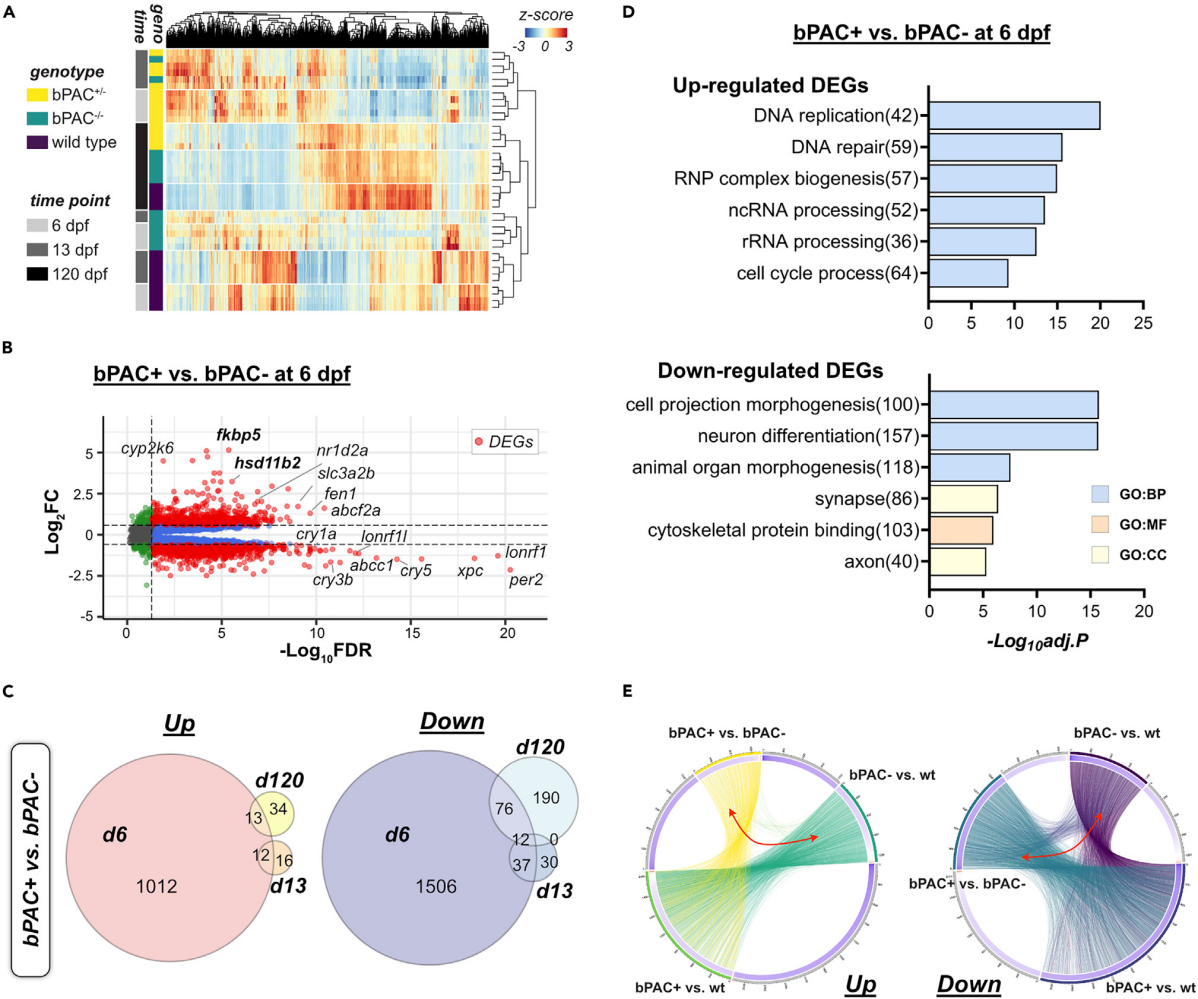
Developmental GC exposure leads to differential gene expressions across the life course (A) Read counts of genes by genotype and time points are represented by *Z* score in the heatmap. Bars on the left side indicate the genotype of fish (yellow: bPAC+, green: bPAC-, dark purple: wild type(wt)) and time points (gray: 6 dpf, dark gray: 13 dpf, black: 120 dpf). (B) Volcano plot showing DEGs (red dots) in bPAC+ and bPAC- brain transcriptome at 6 dpf. Dashed lines indicate FDR and FC cut-off values set at 0.05 and 1.5, respectively. Labels identify the genes associated with cortisol or circadian rhythm among the smallest FDR values. Plots for other time points and comparisons are available in Data S1. A complete list of identified DEGs is described in Table S1. (C) Venn Diagram for up- and down-regulated DEGs among wild type, bPAC- and bPAC+; criteria for DEG identification: |FC|>1.5 and FDR <0.05. (D) Top enriched GO terms for up- and down-regulated DEGs in bPAC+ compared to bPAC- at 6 dpf. A complete list of DEG-enriched GO terms is described in Table S2. (E) Circos plots showing overlapping DEGs by linked line among three pairwise comparisons, bPAC+ vs. bPAC-, bPAC+ vs. wt, bPAC- vs. wt. FDR: False discovery rate, GO: Gene ontology, *adj.p*: adjusted *p*-value.

To identify transcriptional changes caused by hdGC exposure, we performed time-series whole-brain RNA sequencing using larval (6 and 13 dpf) and female adult (120 dpf) brains of bPAC+, bPAC-, and wild-type fish. The global gene expression profiles of bPAC+ were clearly distinct from those of wild types while bPAC- profiles mapped more closely to those of bPAC+ than wild type (Figure S2A). DEGs were identified using the threshold (false discovery rate (FDR) < 0.05 and |log_2_FC (fold-change)| > 1.5) (Figures 3A and 3B, Data S1, Table S1). The overall expression pattern of identified DEGs mainly exhibited a close grouping primarily based on groups and time points except for two 13 dpf bPAC- profiles (Figure 3A). The total number of DEGs identified among different genotypes varied starkly at different time points (Figure 3C). In bPAC+ and bPAC- comparisons, which reveal alterations induced by hdGC exposure, a large number of DEGs were identified at the early larval stage (6 dpf, up/downregulated DEGs: 1037/1631), but a much fewer number of DEGs were detected at 13 dpf (28/79) and 120 dpf (47/278) (Figure 3C). At 6 dpf, we observed transcriptional alterations of genes that were previously reported to be altered following GC treatment, including *fkbp5*^25^ and *hsd11b2*^26^ (Figure 3B, Data S1, Table S1). We did not detect the differential expression of genes in bPAC+ larvae for other known co-chaperons in the GR complex^27^^,^^28^ including *fkbp4*, *hspa1b* (hsp70), *hsp90aa1.1* (hsp90), *ppid* (cyp40), *stip1* (hop), *st13* (hip), and *ptges3a*/*ptges3b* (p23). In contrast, the expression of *hsp90aa1.2* (Log_2_FC: −0.77, FDR: 4.85E-09), *hsp70.1* (Log_2_FC: −0.97, FDR: 0.0005), *hsp70.2*, (Log_2_FC: −1.057, FDR: 4.23E-05), and *hsp70.3*. (Log_2_FC: −0.83, FDR: 0.019) were different in bPAC+ larvae (Table S1). This may suggest that hdGC exposure preferentially regulates some components of GR signaling.

In the adult bPAC+ brains, 27% (13 genes) of the upregulated and 31% (88 genes) of the downregulated DEGs overlapped with DEGs from larval stages (Figure 3C). Differential expression of a subset of identified DEGs was validated in independent biological replicates using real-time qPCR (Figure S3).

Next, we performed GO enrichment tests comparing bPAC+ and bPAC- with the entire set of identified transcripts in our brain sequencing as background genes. Upregulated DEGs in bPAC+ fish brains at 6 dpf were enriched in RNA processing and DNA replication and repair (upper panel of Figure 3D, Table S2). In contrast, downregulated DEGs at 6 dpf were enriched in processes related to nervous system development including neuron differentiation and cell morphogenesis (bottom panel of Figure 3D, Table S2). Next, we performed a gene set enrichment assay to cell-type using published single-cell transcriptome studies for zebrafish brains at 5 dpf (GEO: GSE158142).^29^ Identified DEGs of bPAC+ brains at 6 dpf were enriched in the top marker gene set for progenitors, pharyngeal arch/pectoral fin (mesoderm), and oligodendrocytes (Figure S4, Table S3). Indeed, we confirmed the alteration of neurogenesis in larval bPAC+ fish in a separate report.^16^ Together these results indicate the dysregulation of normal developmental processes in bPAC+ brains in early life, coupled with potential genomic instability caused by replication stress and delayed or immature differentiation of neuronal cells. At 120 dpf, despite a smaller number of DEGs identified (Figure 3C), we noted the significant upregulation of genes involved in pre-mRNA splicing and 5-hydroxymethyl cytosine (hmC) reading (*wdr76*, *neil1*, and *hells*)^30^ suggesting differential epigenetic processes while the downregulation of some of the marker genes for oligodendrocyte (*sox10*, *olig2*) indicating decreased oligodendrocyte differentiation in adult bPAC+ brains (Table S1).

Further, we carried out bPAC+ and wild type or bPAC- and wild type comparisons to determine potential effects resulting from high GC exposure that occurred in the bPAC+ parental generation. Even with more stringent thresholds to identify DEGs (FDR <0.01 and |log2FC| > 2) than those of previous comparisons, the upregulated DEGs enriched GO terms in later developmental stages were similar to those identified in the bPAC+ vs. bPAC- comparison including cell cycle, DNA metabolic process, and RNA processing processes (left panel of Figure S2C). Downregulated DEGs across all time points were enriched in processes related to cell adhesion and cell migration, as well as nervous system development including neuron differentiation, cell morphogenesis, and axon development (right panel of Figure S2C), similar to the bPAC+ versus bPAC- comparison. Also, the identified upregulated DEGs in the larval stage of bPAC+ compared to wild type were enriched in potential metabolic enzymes for GC such as cytochrome monooxygenases, 11β-hydroxysteroid dehydrogenase or sulfotransferases^31^^,^^32^ (left panel of Figure S2C). Some of those DEGs were also significant even in the comparison between bPAC- and wild type (Table S2) suggesting alterations in GC metabolism resulting from elevated GC in parental generation. Lastly, 2.08% (27/1295) and 16.6% (211/1259) of up- or downregulated DEGs identified during larval stages in the comparison between bPAC- and wild type overlapped with DEGs identified in the comparison between bPAC+ and bPAC- (Figure 3E, marked with red double-headed arrows, Figure S2D). However, in adulthood, much fewer overlapping DEGs were identified compared to larval stages (Figure S2D). Together, these results identify transcriptional differences caused by hdGC exposure and suggest the existence of alterations induced by high GC exposure in the parental generation apparent in both bPAC+ and bPAC-.

### High glucocorticoid exposure during development leads to exaggerated transcriptional response to acute stress in adulthood

**Figure 4:**
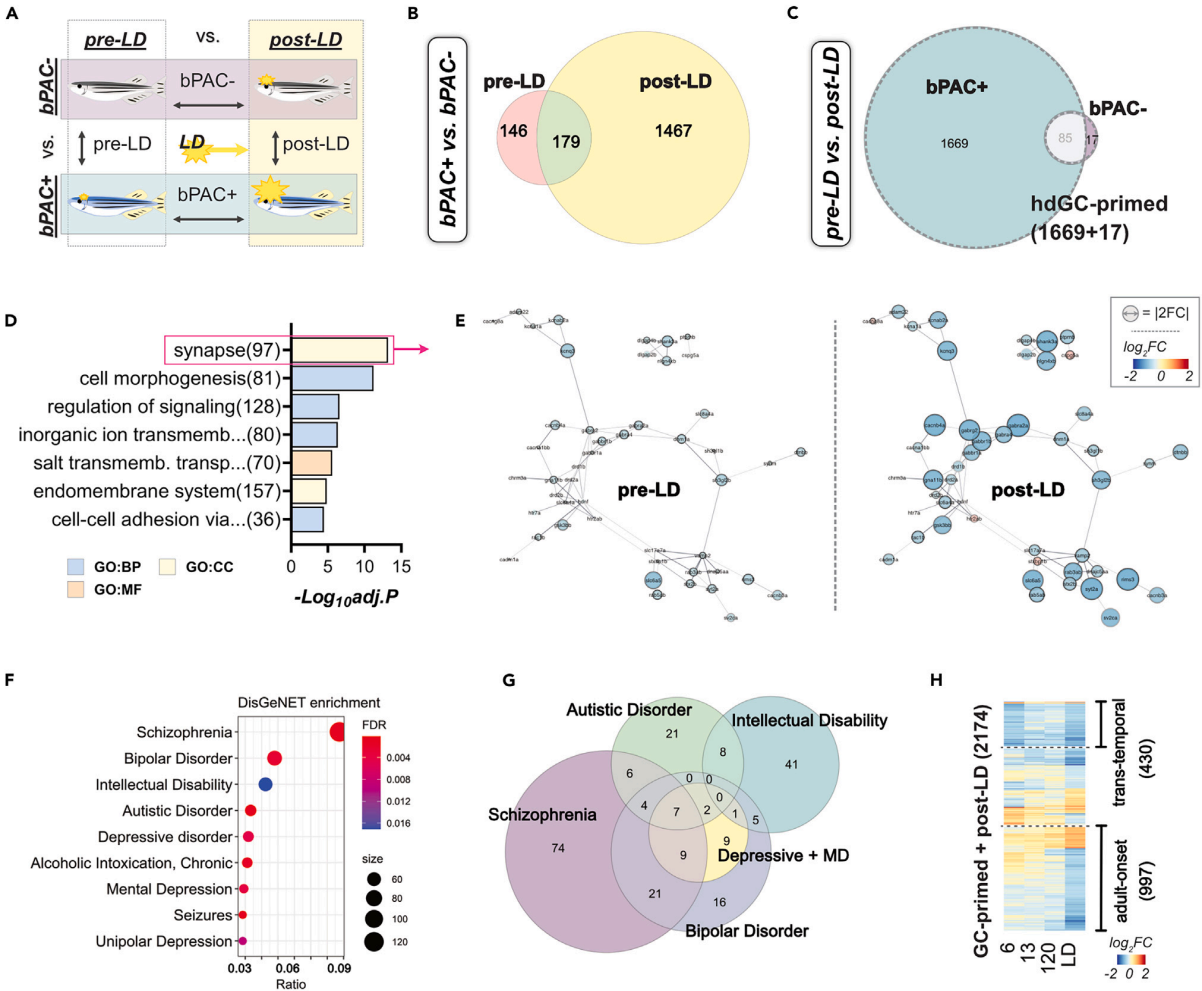
Developmental GC exposure primes differential gene expression following acute stress in bPAC+ adults (A) Schematic for three comparisons to identify transcriptional changes in response to LD exposure in adults. DEGs were determined by FC and FDR. |FC|>1.5 and FDR<0.05. (B) Venn diagram representing DEGs between bPAC+ and bPAC- in pre-LD and post-LD conditions. (C) Venn diagram showing post-LD DEGs from bPAC+ and bPAC-. The list of DEGs is described in Table S3. hdGC-primed genes are marked by a dashed line. (D) The top GO terms enriched in hdGC-primed genes. The enrichment score = -log_10_(adjusted *p*-value). (E) PPI networks of synapse-associated hdGC-primed genes. The size of the node indicates a fold-change. Networks sourced by STRING-db. (F) Result of human disorders enrichment test based on the DisGeNET database using GC-primed genes. The ratio represents overlapping/input genes. Top ranked disorders are Schizophrenia (FDR = 1.12e-04, ratio = 122/883), Bipolar Disorder (FDR = 2.47e-03, ratio = 69/477), Intellectual Disability (FDR = 1.71e-02, ratio = 61/447), Autistic Disorder (FDR = 1.87e-04, ratio = 48/261), Depressive disorder (FDR = 4.51e-03, ratio = 46/289), and Mental Depression (FDR = 4.06e-03, ratio = 42/254). The complete results are noted in Table S5. (G) Venn diagram for hdGC-primed genes that are associated with the top human psychiatric disorders in panel F. (H) Heatmap showing Log_2_FC of 2174 LD-DEGs across time points demonstrated 430 *trans*-temporal and 997 adult-onset genes were mostly downregulated. LD: looming dots, FC: fold change, FDR: False discovery rate, PPI: Protein-protein interaction, ratio: GC-primed genes/disease genes, Depressive + MD: Depressive disorder and Mental Depression.

We next examined the transcriptional response of bPAC+ upon acute stress exposure in adulthood. To identify a class of genes that are induced by acute stress exposure only in bPAC+, we carried out comparisons by genotype (bPAC+ vs. bPAC-) or condition (pre-vs. post-LD) (Figure 4A, Table S4). bPAC+ fish showed a much greater transcriptional response following LD exposure compared to bPAC- fish (Figure 4B). Strikingly, bPAC+ showed more than 17-fold greater number of DEGs in response to LD exposure (bPAC+ in Figure 4C, 1754 genes) compared to bPAC- (bPAC- in Figure 4C, 102 genes). 83% of DEGs induced by LD-exposure in bPAC- were identical to those in bPAC+, while only 17 DEGs were specific to bPAC-. These results indicate that hdGC exposure primed a large number of latent alterations which become only apparent upon acute stress exposure in adulthood in bPAC+ fish. We refer to these differentially regulated genes following LD-exposure in bPAC+ (bPAC+ or bPAC- specific DEGs; 1669 and 17 genes) as hdGC-primed genes (a total of 1686 genes) (Figure 4C, Table S4). hdGC-primed genes were significantly overrepresented in the GO terms such as synapse, cell morphogenesis, and regulation of signaling (Figures 4D and 4E, Table S5), and approximately 80% of hdGC-primed genes (1362 genes) were downregulated. For example, synapse-associated hdGC-primed genes in Figure 3D were mostly downregulated and showed enhanced alteration following LD-exposure (Figure 4E).

Among the genes associated with GC signaling, we observed that the expression of *nr3c1* (GR) is not altered in bPAC+ adults while the expressions of *nr3c2* (mineralocorticoid receptor; MR) and *crhr1* (corticotropin releasing hormone receptor) were significantly downregulated only in bPAC+ adults following LD exposure (Figure S5A). Moreover, we identified that several key molecules within the CRH signaling pathway were altered in adult bPAC+ brains following LD-exposure, consistent with the observed downregulation of *crhr1* in bPAC+ following LD (Figure S5).

### Neuropsychiatric disorder risk genes are over-represented in identified glucocorticoid-primed gene set

Since ELS is a strong risk factor for developing psychiatric disorders in humans which may manifest in adulthood triggered by stressful life events,^1^^,^^2^ we asked whether hdGC-primed genes could overlap with psychiatric disease risk factors identified in humans. Remarkably, human homologs of hdGC-primed genes exhibit a significant enrichment of known psychiatric disease-associated genes curated in the DisGeNET database^33^ (Figure 4F, Table S6). Top-ranked psychiatric disorders were schizophrenia, bipolar disorder, intellectual disability, autistic disorder, and depressive disorder (Figures 4F and 4G). In addition, we carried out a comparison with a recent study that reported 702 GC-primed DEGs linked with long-lasting differentially methylated sites following re-exposure to GC (dexamethasone) in a human hippocampal progenitor/neuron cell line^13^ and identified 103 genes overlapping with DEGs identified in post-LD bPAC+ vs. post-LD bPAC- comparison (up/down = 58/45) and 19 of those were hdGC-primed genes (Figure S6, Table S7). Notably, some of these post-LD bPAC+ DEGs were already modified at 6 dpf in bPAC+ (*trans*-temporal, 430 DEGs), even though the magnitude of alterations were not always maintained at different stages while some others were altered uniquely in bPAC+ adulthood upon acute stress (adult-onset, 997 DEGs) (Figure 4H). These results suggest that some hdGC-primed genes can already be predicted in the early stages following hdGC exposure. Thus, our result identifies a hitherto uncharacterized set of hdGC-primed genes that are overrepresented in synapse and cell signaling molecules as well as homologs of human psychiatric disease risk factors.

### A subset of glucocorticoid-primed transcriptional alterations is associated with differential DNA methylation and alternative splicing patterns in adulthood

**Figure 5:**
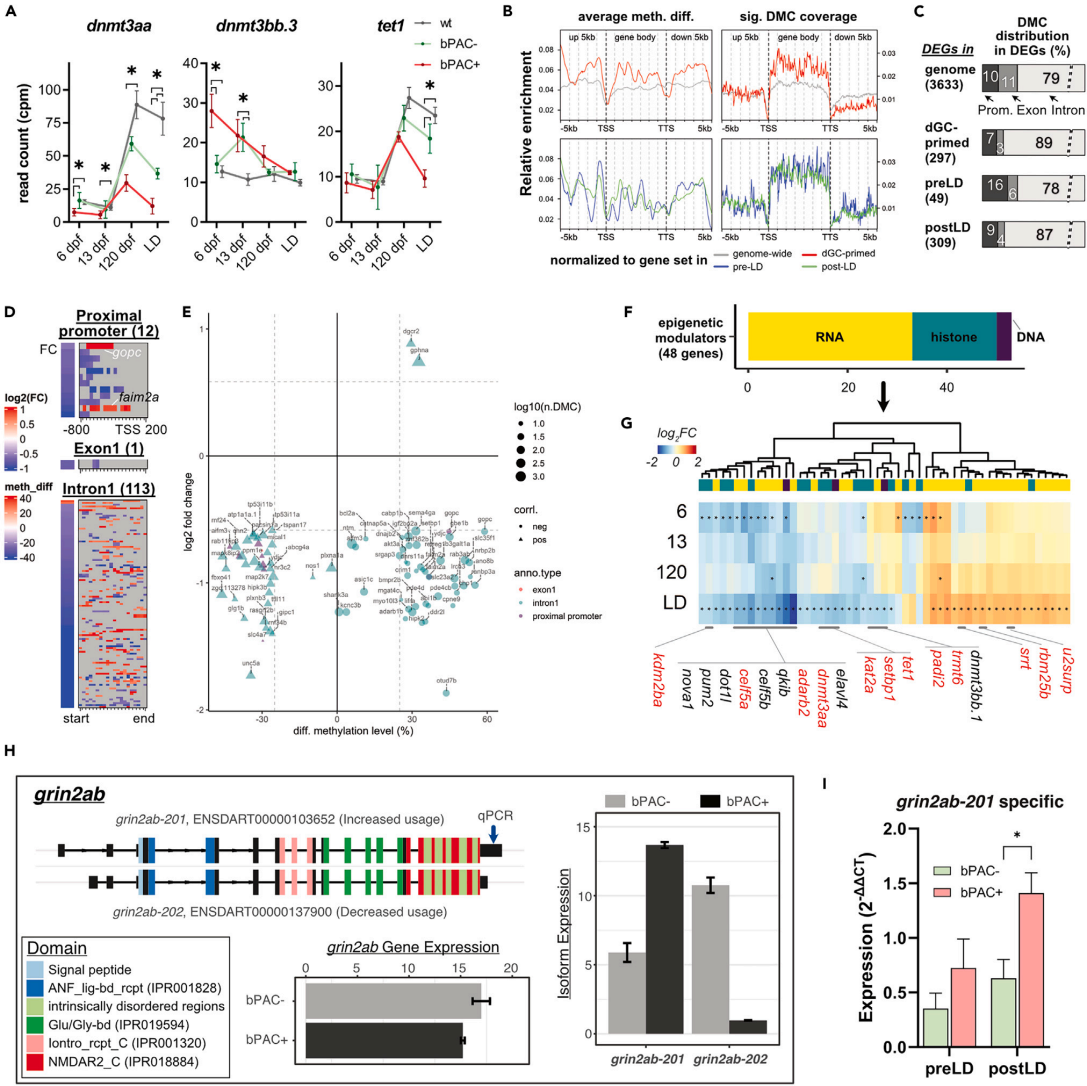
Subsets of GC-primed genes are differentially methylated and spliced in bPAC+ brains (A) Expression trajectories for differentially expressed *dnmt*s in bPAC+ brains. Šídák’s multiple comparisons test followed mixed model ANOVA. The asterisks indicate the time points with significant differential expressions (*adj.p* < 0.05 and |FC| >1.5). *dnmt3aa*: F(6, 39) = 13.13, *p < 0.0001*; *dnmt3bb.3*: F(6, 39) = 11.26, *p < 0.0001*; *tet1*: F(6, 39) = 7.037, *p < 0.0001*. Error bars indicate mean ± SD. wt: wild type. (B) hdGC-primed genes showed a higher ratio of significant DMCs in their gene body regions. meth. diff: methylation level difference. (C) The bar graph depicts the percentage of DEGs, which have DMCs in their proximal promoter, exon, or intron. (D and E) Methylation levels of DMCs on the regulatory regions of genes including proximal promoter, exon1, and intron1 were positively or negatively correlated with subsets of GC-primed genes. (D) Heatmaps present gene expression foldchange and DMCs of genes. (E) x and y axis of the dot plot represent the average methylation level of DMCs in each regulatory region (color of dots), respectively. (F) Bar graph showing known epigenetic regulators associated with DNA (purple, 3 genes), RNA (yellow, 30 genes), and histone (dark green, 15 genes) modification or process among hdGC-primed genes and post-LD DEGs. (G) Heatmap represents log_2_FC of them. The asterisk in each cell refers to FDR <0.05. The red label indicates the existence of DMCs in their corresponding regions. (H) An example of identified differential usage of *grin2ab* transcript variants between bPAC+ vs. bPAC-. Color codes known protein domains. Expression values in bar plots are based on count per million (CPM). The Arrow on the transcript, *grin2ab-201*, indicates primer binding sites for qPCR. (I) Differential usage of the transcript, *grin2ab-201*, was validated by real-time qPCR. Error bars show mean + SEM.

To identify potential mechanisms establishing hdGC-primed transcriptional alterations, we analyzed expression patterns of DNA methylation modifiers across different time points (Figure S7). Among them, we found a significant difference in the levels of *dnmt3aa* and *dnmt3bb.3* in the bPAC+ fish compared to bPAC- and wild type at 6 dpf (Figure 5A). A significant difference in *dnmt3aa* level in bPAC+ was maintained into adulthood. Similarly, the level of *tet1,* implicated in demethylation, is altered in bPAC+ adults compared to bPAC- and wild type after LD exposure (Figure 5A).

To determine whether differential DNA methylation contributes to the alteration of hdGC-primed gene expression following LD exposure, we identified methylated cytosines (5mCs) in the adult brains of bPAC+ before LD exposure using long-read whole genome sequencing via the Oxford nanopore sequencing platform^34^^,^^35^ (Figure S8). Since the majority of hdGC-primed genes defined here (1657/1686 hdGC-primed genes) are differentially regulated in bPAC+ compared to both wild type and bPAC- (Figure S8A) and previously published reports described transgenerational effects on DNA methylation in zebrafish,^36^^,^^37^ we carried out a comparison of bPAC+ with wild-type brain to identify differentially methylated CpG sites (DMCs). We globally identified significant differentially methylated CpG sites (DMCs), which met the criteria of exhibiting more than a 25% difference in mean methylation level with an FDR below 0.05. These DMCs exhibited a distribution across various genomic regions, including exonic regions (comprising 1% of hypomethylated and 1% of hypermethylated CpGs of the total), intronic regions (encompassing 51% of hypomethylated and 53% of hypermethylated CpGs of the total), and potential proximal promoter regions (extending 800 bp upstream and 200 bp downstream of the transcription start site (TSS), accounting for 2% of hypomethylated and 3% of hypermethylated CpGs of the total) (Figures S8B–S8E).

Initially, we prioritized DMCs situated on gene bodies and proximal regions of transcription start sites (TSSs), which possess regulatory potential (Figures S9A and S9B). In the hdGC-primed gene set, significant DMCs were notably enriched within the gene bodies compared to across the genome (upper panels of Figures 5B and S9A). At the gene level, 89% and 3% of hdGC-primed genes contain DMCs within their intronic and exonic regions, respectively (Figure 5C). Interestingly, the occurrence of significant DMCs in the proximal flanking regions of both TSSs and transcription termination sites (TTSs) of hdGC-primed genes was observed to be lower compared to the genome-wide average (Figures S9C and S9D). These results suggest that impacts on hdGC exposure are more pronounced in intronic regions than proximal promoter regions. Therefore, in this study, we focused on DMCs within proximal promoters, exon1, and intron1 which were reported as the regions showing a relatively consistent correlation between DNA methylation and gene expression.^38^^,^^39^ While the observed DMC ratio within proximal promoters was lower than those in intron, we identified significant DMCs that were positively or negatively correlated with their average methylation level and gene expression in 9 proximal promoters (negative/positive: 2/7), one exon1 (positive), and 77 intron 1 (44/33) regions (Figures 5D and 5E, Table S8). Enriched patterns of DMCs in intron1 were not detectable, but DMCs within proximal promoters were mainly located upstream of TSS (Figure 5D). Moreover, average methylation levels of DMCs within the proximal promoter of two membrane protein genes, *gopc* and *faima2a*, were negatively correlated with their gene expression following LD exposure (Figures 5D and 5E). We observed genes associated with gene expression regulators (*hipk2*, *ccnt1*) as well as top two GO terms in Figure 4D (synapse and cell morphogenesis; *gphna*, *plxnb2b*, *sema5ba*, and *unc5a*) (Figure 5E, Table S8). In addition, we found hypermethylated CpGs in genomic regions associated with hdGC-primed genes, *auts2a*, *mapre3a*, *igfbp5a*, and *pde4cb*, which overlapped with identified GC-primed DEGs in *in vitro* dexamethasone-treated human neurons^13^ (Table S8). Overall, these results suggest that altered DNA methylation patterns may be associated with the regulation of the expression of a subset of hdGC-primed genes in bPAC+ brains.

Lastly, since we observed the enrichment of DEGs in adult brains (120 dpf) in functions of RNA processing processes (Figure S2C), we analyzed the expression pattern of a broader category of epigenetic modifiers in hdGC-primed genes and DEGs post-LD-exposure. A total of 48 identified genes were associated with RNA processing including splicing and modification (yellow bar in Figure 5F, up/down = 18/12), histone modification (dark green bar in Figure 5F, up/down = 2/13), and DNA methylation modification (purple bar in Figure 5F, up/down = 0/3) which were altered following LD-exposure in bPAC+ brain (Table S9). Some of them were differentially expressed in the 9-month-old brains as well following LD exposure (Figure S10). Moreover, we identified clusters of significant DMCs including at least 3 DMCs in a 100bp window, on proximal promoter and gene body regions of 12 genes out of those 48 genes, whose methylation patterns were correlated with their expression pattern following LD-exposure positively or negatively (labeled as red in Figures 5G and S11). Strikingly 62% of altered epigenetic modifier genes we identified (48 genes) were associated with RNA processing including that may play important roles in the nervous system.^40^ Indeed, we identified differential usage of transcript variants from 6 genes between bPAC+ and bPAC- brains following the LD-exposure from our RNA-seq data (Table S10). For example, we identified differential usage of isoforms of one of the glutamate receptor genes, *grin2ab*, and confirmed more usage of the transcript, *grin2ab-201* (Ensembl: ENSDART00000103652), than *grin2ab-202* (Ensembl: ENSDART00000137900) in bPAC+ brains following LD-exposure by real-time qPCR (Figures 5H and 5I). However, there was no difference in their protein-coding regions between these two isoforms (Figure 5H). Similarly, we found significant differences in functional domain coding regions of genes *kmt2e*, *zgc:123105* (ortholog to human *KIAA1191*), *prrc2a*, and *scg2b* for their intrinsically disordered regions (IDRs), but no significant differences in the inclusion of functional domains (Figure S12). Altogether, our results suggest that hdGC exposure can affect adult brains in a long-term manner by priming epigenetic modulators.

## Discussion

We report here that hdGC exposure led to exaggerated endocrine stress responses and primed a set of genes to exhibit altered transcriptomic responses to acute stress in adulthood. We revealed that a gene set enriched in the processes of DNA/RNA processing, neuron projection, and neuronal signaling components is altered following hdGC exposure. We identified a comprehensive list of a brain-wide hdGC-primed gene set and revealed that some of these DEGs are associated with neuropsychiatric disorders in humans and include a set of gene expression modulators. Lastly, we showed DNA methylation and RNA processing as gene regulatory mechanisms that may contribute to establishing the hdGC-primed response to acute stress in adulthood.

The hdGC-primed gene set identified here shares a significant overlap with a previous report of GC-primed genes using neuronal cells derived from the human hippocampal progenitor cell (HPC) line.^13^ However, we could only confirm a limited number of overlapping DNA methylation pattern alterations between our study and those identified in the previously published studies. DNA methylation patterns and gene expression are known to vary in the tissue/cell type and species-specific manner.^39^^,^^41^^,^^42^^,^^43^ Given the possible roles and transcriptomic alterations in oligodendrocytes and astrocytes following stress and excess GC,^44^^,^^45^ major differences between our and the previously reported GC-primed genes likely result from differences in the type of samples used namely *in vitro* human neurons versus zebrafish whole-brain samples containing neuronal and non-neuronal cells.

Our results indicate that LD-exposure alters *nr3c2* (MR) and *crhr1* expression specifically in hdGC-exposed animals, while the expression of *nr3c1* (GR) is not altered. While GCs can bind both GR and MR in the brain, where many cells express both types of receptors, MR binding typically occurs at low levels of GC concentration, such as during resting state. In contrast, the lower affinity GR is usually occupied only under high GC concentration, such as under stress or at the circadian peak.^46^ Accordingly, MR and GR operate coordinately in a complementary fashion in response to environmental demands and an imbalance of the MR/GR ratio is predicted to compromise the initiation and termination of the stress response leading to HPA axis dysregulation and impaired behavioral adaptation.^47^^,^^48^^,^^49^ In this regard, it will be interesting to determine the specific site of MR:GR expression imbalance upon LD exposure in our model and assess their potential roles in affected behaviors in bPAC+ animals.

In this study, significant differences in the expression of known co-chaperone genes within the GR complex were not observed following LD-exposure. However, differential expression patterns were noted in certain co-chaperon genes including *hsp90aa1.2, hsp70.1, hsp70.2*, and *hsp70.3* at 6 dpf (Table S1). Although the absence of a difference in the expression level observed in another duplicated hsp90aa1 gene (*hsp90aa1.1*) and the generally low expression of *hsp70* genes were detected, such findings in our whole-brain bulk RNA-seq could potentially come from regional or cell-type specific expression or regulation of those genes. In the same context, further regional/cell-type specific studies may reveal the differences in other co-chaperons which did not show the differential expression in our bulk RNA-seq result.

As early exposure to excess GC is considered a global risk factor for the development of mental disorders, our behavioral pipeline was tailored to assess performance in key behavioral domains rather than disease-specific tasks. bPAC+ fish did not show defects in aspects of perception as they can recognize visual and olfactory stimuli in the form of social cues and food pellets. Interestingly, bPAC+ fish exhibited a reduction in the maintenance of social interactions compared to the wild type, but no statistically significant difference was detected to bPAC-. We hypothesize that the attenuation of social interactions could potentially be influenced by paternal epigenetic memories resulting from developmental exposure to GCs. These effects should not be underestimated as they could serve as a significant contributing factor to the emergence of divergent social interaction patterns in adulthood. In previous zebrafish ELS studies, diminished shoaling behavior was observed following early-life social isolation and was associated with both motivational deficiencies linked to dopaminergic systems^50^ and increased sensitivity to stimuli which was reversed by anxiolytic treatment.^51^ Impairments in social behavior or social withdrawal are common among different psychiatric disorders, including autism^52^ and schizophrenia,^53^ and have been described in rodent models of ELS in which deficits in social interaction in the absence of social recognition and approach have been observed.^54^ Further, bPAC+ fish showed deficits in fear learning compared to wild type using a Pavlovian conditioning paradigm that pairs electric shock with the color green. It is possible that perceived salience of electric shock as a stressor rather than fear learning is diminished in bPAC+. However, we consider this possibility less likely as bPAC+ can respond robustly to acute stress presented in the form of LD. This phenotype is consistent with what has been reported in rodent ELS models, which showed deficits in fear learning, learning impairment, and decreased cognitive flexibility.^55^^,^^56^^,^^57^ Differences in how zebrafish learn fear association are dependent on whether they are reactive or proactive responders to stress, raising the question of how bPAC+ fish’s response fits into that spectrum.^58^

Recently, gene catalogs of neuropsychiatric disease-associated genes following stressful events including early life stress were reported based on gene expression networks, eQTLs, and GWAS.^33^^,^^59^^,^^60^ The majority of hdGC-primed genes that are associated with schizophrenia, bipolar disorder, depressive disorder, and autistic disorder-associated showed an exaggerated transcriptional response to stress in adult bPAC+ brains. Those DEGs were overrepresented in synaptic signaling processes including genes for GABA receptors (*gabrg2*, *gabbr1a*, *gabbr1b*, *gabra1*, *gabra2a*, *gabra4*, *gabrg2*), glutamate receptors and transporter (*grm1b*, *grm3*, *grm5b*, *grik3*, *grin2b*, *slc17a7a*), dopamine receptors (*drd2a*), cholinergic receptor (*chrm2a*), opioid receptor (*pnoca*), and neurexins (*nlgn1*, *nlgn4xb*). In a human study, genetic variants associated with GR-mediated immediate transcript response were able to predict risk for psychiatric disorders including depression.^61^ Consistent with this, our findings suggest developmental GC exposure leads to differences in GR-induced transcriptional activation in adult brains and may mediate the risk for psychiatric disorders by altering a network of neuronal signaling-related stress-sensitive genes.

Interestingly, we identified a large set of upregulated DEGs that were enriched in DNA metabolism, various RNA processing processes, and posttranslational modifications as well as epigenetic modifiers including DNA methyltransferases. For example, we identified a subset of upregulated hdGC-primed RNA splicing factors that are involved in U1, U2, or U12-dependent mRNA splicing, including *rbmx2, rbm5, srrm1, rbm25a/b, luc7l,* and *rnpc3*.^62^^,^^63^^,^^64^^,^^65^ Also, we observed a downregulation of genes encoding hdGC-primed RNA-binding proteins, such as *elavl4, qkib, celf2, celf5a, celf5b, nova1,* and *pum2*, which may play important roles in the nervous system.^66^^,^^67^^,^^68^^,^^69^ In the context of tissue-dependent exon usage, it was reported that the alternative transcription start and termination sites played an important role.^70^ Therefore, the identified differential usage of transcript variants may be regional or cell-type dependent alterations, suggesting that hdGC exposure may be able to prime genes in a cell-type or regional-specific manner. In this study, we observed no significant differences in the inclusion of functional domains of differentially used transcript variants (Figures 5H and S12) and differential expression of processed non-coding transcript (*zswim8-202*, Ensembl: ENSDART00000100965) (Figure S12). These results suggest that alternative splicing events can be induced in adult brains following hdGC exposure even though more experiments are required to reveal the biological meaning of differential usage of identified isoforms. Furthermore, alternative donor sites are involved in exon definition,^71^ repressor-activator competition^72^ as well as psychiatric disorders.^73^^,^^74^ Since an increasing body of evidence underscores the role of alternative splicing in diseases including psychiatric disorders,^73^^,^^75^ further studies using long-read sequencing may reveal an even greater number of alternative splicing events induced by exposure to hdGC. Collectively, hdGC exposure may increase the risk of the dysregulation of gene expression in the later life stage by altering epigenetic regulations and RNA processing in adulthood.

In conclusion, we identified highly exaggerated transcriptional response in animals with exposure to a high level of GC during development. However, further investigation is required to reveal the consequence of altered epigenetic modulators and a direct mechanistic link between the identified hdGC-primed genes and alterations in regulatory mechanisms.

We propose that the brain DEG set identified here will be useful for guiding future studies dissecting the mechanisms underlying developmental GC exposure and alteration of behaviors and neuropsychiatric disease susceptibility in adulthood. The zebrafish model presented here provides an opportunity to dissect the long-term effects of endogenous GC elevation during development and can be leveraged in future studies to identify the critical period, intensity, and duration of developmental GC exposure as well as epigenetic regulatory mechanisms including RNA processing and DNA methylation that leads to adult dysfunction.

### Limitations of the study

Our “double-hit” transgenic model is raised under ambient light condition which elevates GC throughout its development. Therefore, we cannot discern the critical window of sensitivity for the effects of GC. Also, among the DEGs identified, we cannot distinguish those that are directly regulated by GC exposure. Future studies aimed at limiting the duration and window of optogenetic GC elevation can identify direct targets of hdGC. Another limitation is the use of bulk RNA sequencing. While, we attempted to analyze cell-type-specific differences using published scRNA sequencing, future studies using defined cell types is essential to understand complex cell-type specific effects of GC.

## STAR★Methods

### Key resources table

**Table:** 

REAGENT or RESOURCE	SOURCE	IDENTIFIER
**Chemicals, peptides, and recombinant proteins**		

Ethyl acetate	Sigma-Aldrich	Cat#270989
Ethanol, BioUltra	Sigma-Aldrich	Cat#51976
2-Propanol	Sigma-Aldrich	Cat#59304
Nuclease-Free Water	Invitrogen™	Cat#AM9937
GlycoBlue™ Coprecipitant	Invitrogen™	AM9516
Phosphate buffered saline	Sigma-Aldrich	Cat#P4417

**Critical commercial assays**		

HTRF® Cortisol Kit	Cisbio	Cat# 62CRTPEG
RNAlater™ Stabilization Solution	Invitrogen™	Cat#AM7021
Quick-RNA miniprep Kit	Zymo Research	Cat#R1055
High-Capacity RNA-to-cDNA™ Kit	Applied Biosystems	Cat#4387406
PowerUp™ SYBR™ Green Master Mix	Applied Biosystems	Cat#A25778
RNA 6000 Nano kit	Agilent	Part#5067-1511
TruSeq Stranded mRNA Library Prep kit	Illumina	Cat#20020595
DNeasy® Blood & Tissue kit	QIAGENE	Cat#69504
Ligation Sequencing kit	Oxford Nanopore Technologies Ltd.	Cat#SQK-LSK114
PromethION Flow Cell Packs (R10.4.1)	Oxford Nanopore Technologies Ltd.	Cat#FLO-PRO114M

**Deposited data**		

Time-course whole brain transcriptome and methylome profiles of early life high GC exposed zebrafish	European Nucleotide Archive (ENA)	ENA: PRJEB53713
single-cell object: GSE158142_zf5dpf_cc_filt.cluster.rds	Raj et al.^29^; Gene Expression Omnibus (GEO)	GEO: GSE158142
List of significantly regulated acute only transcripts that map to a long-lasing DMS (*n* = 254).	Provençal et al.^13^	Dataset_S01:S06

**Experimental models: Organisms/strains**		

Zebrafish:*Tg(star:bPAC-2A-tdTomato)*^*uex300+/−*^	Ryu Lab	ZFIN: uex300
Zebrafish: Tübingen strain	Ryu Lab	

**Oligonucleotides**		

Primers for qPCR	See Table S11	

**Software and algorithms**		

MinKNOW (v22.12.05)	Oxford Nanopore Technologies Ltd.	N/A
FASTQC	GitHub	https://github.com/s-andrews/FastQC
fastp (v0.23.4)	GitHub	https://github.com/OpenGene/fastp
HISAT2 (v2.1.1)	GitHub	http://daehwankimlab.github.io/hisat2/
Stringtie (v1.3.3)	GitHub	https://github.com/gpertea/stringtie
edgeR (v3.42.4)	Bioconductor	https://git.bioconductor.org/packages/edgeR
R (v4.3.1)	r-project	https://cran.r-project.org/
Rstudio (2023.12.1 Build 402)	posit	https://posit.co/download/rstudio-desktop/
clusterprofiler (v4.8.3)	Bioconductor	https://git.bioconductor.org/packages/clusterProfiler
gProfiler2 -- an R package(v0.2.3)	r-project	https://CRAN.R-project.org/package=gprofiler2
DisGeNET (v0.99.3)	GitHub	https://github.com/jinfar/disgenet2r
Seurat (v5.0.3)	GitHub	https://github.com/satijalab/seurat
String-db (v11.0b)	STRING CONSORTIUM	https://string-db.org/
Cytoscape (v3.8.2)	Cytoscape	https://cytoscape.org/
IsoformSwitchAnalyzeR (v2.0.1)	Bioconductor	https://git.bioconductor.org/packages/IsoformSwitchAnalyzeR
pycoQC	GitHub	https://github.com/a-slide/pycoQC
Guppy (v6.4.2)	Oxford Nanopore Technologies Ltd	N/A
minimap2	Oxford Nanopore Technologies Ltd	https://github.com/lh3/minimap2
modbam2bed	Oxford Nanopore Technologies Ltd	https://github.com/epi2me-labs/modbam2bed
DSS (v2.50.1)	Bioconductor	https://git.bioconductor.org/packages/DSS
Genomic Ranges (v1.54)	Bioconductor	https://git.bioconductor.org/packages/GenomicRanges
deeptools (v3.3.1)	Max Planck Institute for Immunobiology and Epigenetics, Freiburg	https://deeptools.readthedocs.io/en/develop/
EnrichedHeatmap (v1.32)	Bioconductor	https://git.bioconductor.org/packages/EnrichedHeatmap
Integrative Genomics Viewer (IGV) desktop application (v2.16.2)	Broad Institute	https://github.com/igvteam/igv
Prism (v10.2.0 (392))	Graphpad Software Inc.	N/A
scripts for bioinformatic analysis	This study	https://github.com/minkechoi/tx_star-bPAC_brain

**Other**		

Step-wise protocols	protocols.io	https://doi.org/10.17504/protocols.io.kxygx9ooog8j/v3

### Resource availability

#### Lead contact

Further information and requests for resources should be directed to and will be fulfilled by the lead contact, Soojin Ryu (s.ryu@exeter.ac.uk).

#### Materials availability

This study did not generate new unique reagents.

#### Data and code availability

• All sequenced reads for RNA-seq have been deposited at European Nucleotide Archive (ENA: PRJEB53713) and are publicly available. Accession numbers are listed in the key resources table. The single-cell RNA-seq object “GSE158142_zf5dpf_cc_filt.cluster.rds” was downloaded from NCBI GEO: GSE158142 (Raj et al., 2020). List of DEGs that map to a long-lasing DMS (n = 254) was downloaded from Dataset_S01:S06 of Provençal et al., 2019.
• The detailed data processing and scripts for bioinformatic analysis are available at the GitHub repository. The address of the link is listed in the key resources table.
• Any additional information required to reanalyze the data reported in this paper is available from the lead contact upon request.

### Experimental model and study participant details

All animal procedures were carried out in compliance with the ethical guidelines of the national animal welfare law and approved by relevant authorities (Landesuntersuchungsamt Rheinland-Pfalz, Germany, Project number 23 177-07/G20-1-033 or UK Home Office PPL number PEF291C4D).

#### Number of experimental animals used

For the larval cortisol assays, a total 192, 168, 144, 144, 144, 72, 72, 54, and 25 larvae were pooled into 6 samples per group (wild type and bPAC+) were used at 0, 1, 2, 3, 4, 5, 6, 9, and 13 dpf, respectively (Figure 1D). For the cortisol assays at later stages, 6 fish at 1 mpf in each group (wild type and bPAC+), 6, 19, 12, 12, 18 wild type and 6, 18, 14, 12, 18 bPAC+ at 1, 2, 4, 8, 16 mpf, respectively were used (Figure 1E). In addition, 54 fish were used for cortisol assay following LD-exposure (Figure 1G). For the behavior analysis (Figure 2), a total of 240 female adult zebrafish between 6 and 9 months old were tested. For the RNA-seq, a total of 300 zebrafish larvae at 6 dpf and 13 dpf per group (TU, bPAC-, bPAC+), and 30 adult female zebrafish at 120 dpf per group (TU, bPAC-, bPAC+) were used (Figures 3 and 4). For the whole-brain DNA isolation, 6 wild type and 6 bPAC+ adult brains at 9 months old were used.

#### Zebrafish husbandry and maintenance

The tübingen (TU) strain and transgenic line, *Tg(star:bPAC-2A-tdTomato)*^*uex300*^ were kept at 28°C on a 12:12 h light/dark cycle and housed at a maximum density of 5 fish/L. For experiments with adult fish, only females were used.

### Method details

#### Sample collection

All samples were collected within a 2-h window in the morning (08:30 to 10:30) prior to daily feeding.

#### Whole-body cortisol assay

The competitive cortisol assay (HTRF Cortisol Kit, Cisbio, Codolet, France) was performed following the manufacturer’s protocol. ELISA signal was detected by CLARIO star plate reader (BMG Labtech, Ortenberg, Germany).

#### Behavior tests

Social behavior tests and acute stressor delivery using looming dot (LD) presentation were carried out as described in Cook et al*.*^20^ Basal locomotion, food consumption, and fear conditioning assay were performed following the protocol in online methods.^76^ For all behavioral tests, female adult zebrafish (6–9 months old) were moved into a designated behavior experimental room one week prior to testing and housed in groups of 10 fish in 3L tanks.

#### RNA preparation and sequencing

Each sample for the larval stage (6 and 13 dpf) contained 25–30 larval whole-brains whilst for the adult stage (120 dpf) three brains constituted one sample. We dissected the larval whole-brains (at 6 or 13 dpf) in the RNAlater (Invitrogen, Carlsbad, CA, USA) after overnight incubation and the adult brains (at 120 dpf) were dissected in PBS on ice and then snap frozen in liquid nitrogen. Samples were kept at −80°C until further processing. Larval and adult samples were completely homogenized in RNA Lysis buffer from the Quick-RNA miniprep Kit (Zymo Research, Irvine, CA, USA) using a pestle and micro-tube homogenizer (for 30 s) or TissueLyser LT (QIAGEN, Dusseldorf, Germany) at 25 Hz for 1 min and then 15 Hz for 2 min, respectively. Total RNAs were isolated by following the manufacturer’s protocol and kept at −80°C until further use. Transcripts were quantified using real-time qPCR and next-generation mRNA sequencing. cDNA was synthesized using a High-Capacity RNA-to-cDNA Kit (Applied Biosystems, Waltham, MA, USA) following the manufacturer’s protocol. The real-time qPCR was performed with PowerUp SYBR Green Master Mix (Applied Biosystems, Waltham, MA, USA) and specific primers. Primer information for qPCR is described in Table S11. mRNA-seq library preparation and sequencing were performed by TRON gGmbH (Mainz, Germany) using the Illumina NovaSeq6000 platform. Briefly, paired-end TruSeq Stranded mRNA libraries (Illumina, San Diego CA, USA) were constructed and sequenced for over 20M of 50 bp reads/sample. A total of 60 samples was sequenced, consisting of 5 biological replicates at four different time points (6, 13, 120 dpf, and acute-stressed at 120 dpf) for each genotype (wild type, bPAC+ and bPAC-).

#### DNA preparation and sequencing

Whole-brains were collected from 9-month-old bPAC+ and wildtype fish. 6 individual brains per group were used for DNA isolation using DNeasy Blood & Tissue kit (QIAGENE, Hilden, Germany) following the manufacturer’s protocol. Purified DNA was sequenced using the Oxford nanopore sequencing system. Ligation sequencing libraries were prepared from 1000 ng DNA using the Ligation Sequencing kit (Oxford Nanopore Technologies Ltd., Oxford, UK) following the manufacturer’s protocol. The clean-up step after adapter ligation was intended to size-select fragments and was carried out using a Long Fragment Buffer. The library was loaded on a single R10.4.1 (FLO-PRO114M) flow cell and sequenced on a PromethION 24 device within 72 h. MinKNOW (v22.12.05) was used to supervise the initial sequencing run.

#### Bioinformatic analyses

##### mRNA sequencing and analyses

RNA sequencing analyses were performed following online methods.^76^ Briefly, processed qualified sequencing reads by FASTQC,^77^ fastp^78^ were mapped to the zebrafish reference genome assembly (GRCz11) with Ensembl annotation version 107 using HISAT2.^79^ We estimated the expression of transcripts using Stringtie^80^ and DEGs using edgeR.^81^ Downstream and statistical analyses were performed with in-house R scripts on Rstudio.^82^ DEGs were defined with criteria; |FC| > 1.5 and FDR <0.05 for the comparisons between bPAC+ vs. bPAC-, or |FC| > 2 and FDR <0.01 for the comparisons between bPAC+ vs. wild type. Functional analyses were performed with the R packages including clusterprofiler,^83^ gProfiler2,^84^ enrichGO,^83^ and disgenet2r.^33^ To perform cell-type enrichment test, the single-cell RNA-seq object “GSE158142_zf5dpf_cc_filt.cluster.rds”^29^ was downloaded from NCBI GEO. Identified the top 250 markers for each cluster using FindAllMarkers function from the Seurat_5.0.3 package^85^ were used for creating custom gene sets for GSEA. The enrichplot_1.20.3 package^83^ was used to create enrichment plots using gseaplot2 function. Protein-protein interaction networks were constructed and visualized by using the String database^86^ and Cytoscape.^87^ Alternative splicing and isoform switches were analyzed by using IsoformSwitchAnalyzeR.^88^ We used the following threshold and criteria to identify differential usage of isoforms; expression of isoform more than 10 in at least one of the groups, gene switch FDR <0.05, |differential isoform fraction (dIF)| > 0.4, and known domain identified isoforms only.

##### Long read sequencing

The sequenced reads quality assessment was done using pycoQC.^89^ The reads from each sample were base-called using Guppy (v6.4.2, Oxford Nanopore Technologies Ltd.) and mapped to the *Danio rerio* (GRCz11) genome using minimap2 aligner.^90^ 5mC CpG modified bases were determined using the modbam2bed tool (Oxford Nanopore Technologies Ltd).

##### Differential methylation analysis

Differential methylation analysis between control and stress samples was performed using DSS Bioconductor package^91^ with *p* < 0.05. Bases were considered as differentially methylated if FDR <0.05 and the absolute DNA methylation difference between the two groups was larger than 25%. Genomic coordinates of the exon, intron, proximal promoter, and intergenic region for *Danio rerio* genome (GRCz11) were obtained from the UCSC genome browser. Proximal promoters were defined as upstream 800bps and downstream 200bps from TSS. Differentially methylated cytosines were annotated based on genomic positions into exons, introns, proximal promoters, and intergenic regions using the findOverlaps function of GenomicRanges package.^92^ In the downstream analyses associated with significant DMCs, we used DMCs which have more than 10 reads coverage. Methylation browser tracks were created using deeptools bamCoverage utility.^93^ Visualization of DMCs was achieved by using the R package EnrichedHeatmap (v3.18)^94^ and Integrative Genomics Viewer (IGV).^95^

### Quantification and statistical analysis

Statistical analyses were performed using R and Prism 9 (Graphpad Software Inc, San Diego, CA, USA). Before testing for statistically significant differences between groups, data were tested for normality and variance. We used unpaired t-tests (two-tailed) or Mann-Whitney tests for two-group comparisons, ordinary one-way ANOVAs, or two-way repeated measures (RM) ANOVA (mixed model ANOVA) with Šídák’s or Dunn’s or Tukey’s multiple comparisons test as a post-hoc analysis for more than two groups, and built-in statistical adjustments in the R packages for multiple comparisons including False Discovery Rate (FDR). Log rank test was used for the social approach comparisons.

### Additional resources

The detailed protocol for generating transgenic animal, behavioral tests in this study is available as stepwise online methods.^76^

## Supplemental information

Document S1. Figures S1–S12

Data S1. Volcano plots for all pairwise comparisons (|FC|>1.5 and FDR<0.05), related to Figure 3B
