# Supplementary material for: Exposure to elevated glucocorticoid during development primes altered transcriptional responses to acute stress in adulthood
Source: iScience. 2024 May 31;27(7):110160. doi: 10.1016/j.isci.2024.110160 (PMC11233911; doi:10.1016/j.isci.2024.110160)
Supplement: Document S1. Figures S1–S12 [file mmc1.pdf]

iScience, Volume *27*

Supplemental information

Exposure to elevated glucocorticoid during development primes altered transcriptional responses to acute stress in adulthood

Min-Kyeung Choi, Alexander Cook, Kanak Mungikar, Helen Eachus, Anna Tochwin, Matthias Linke, Susanne Gerber, and Soojin Ryu

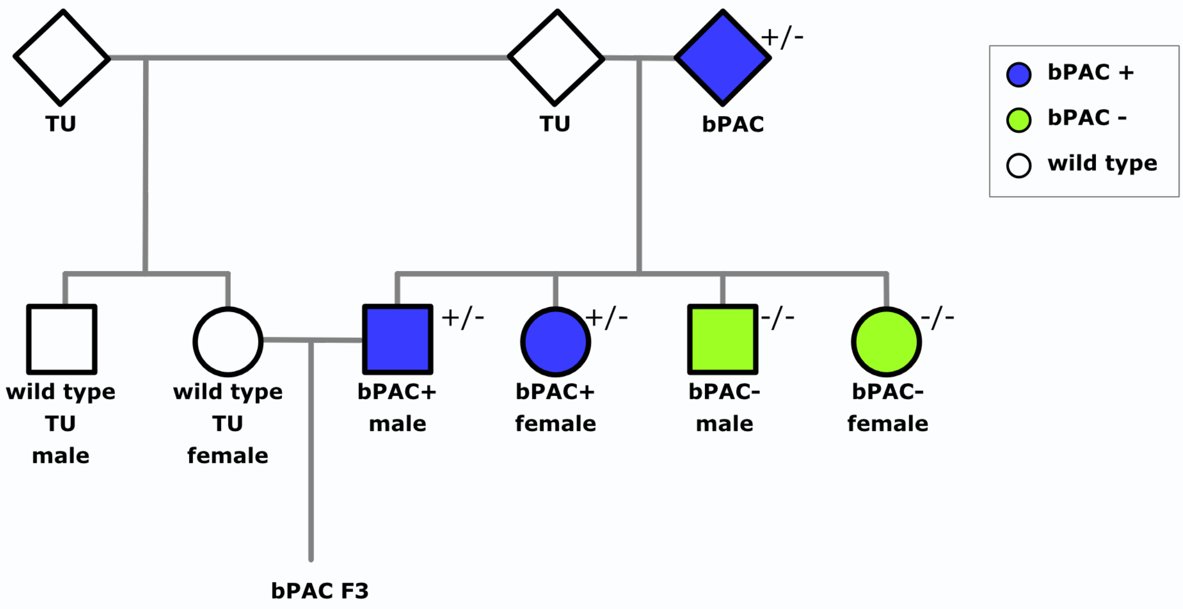

Figure S1. Animal crossing scheme (related to Figures 1-3). bPAC+ transgenic fish line was maintained by crossing heterozygous bPAC+ with wild type (TU). bPAC+ and bPAC- larvae were screened based on the presence (for bPAC+) or absence (for bPAC-) of tdTomato signal in the interrenal gland at 4 days post-fertilization (dpf). However, for cortisol measurements during early developmental stages prior to the onset of the tdTomato expression, we utilized progenies of homozygous bPAC+ crossed to wildtypes. Adult brain samples were collected from females.

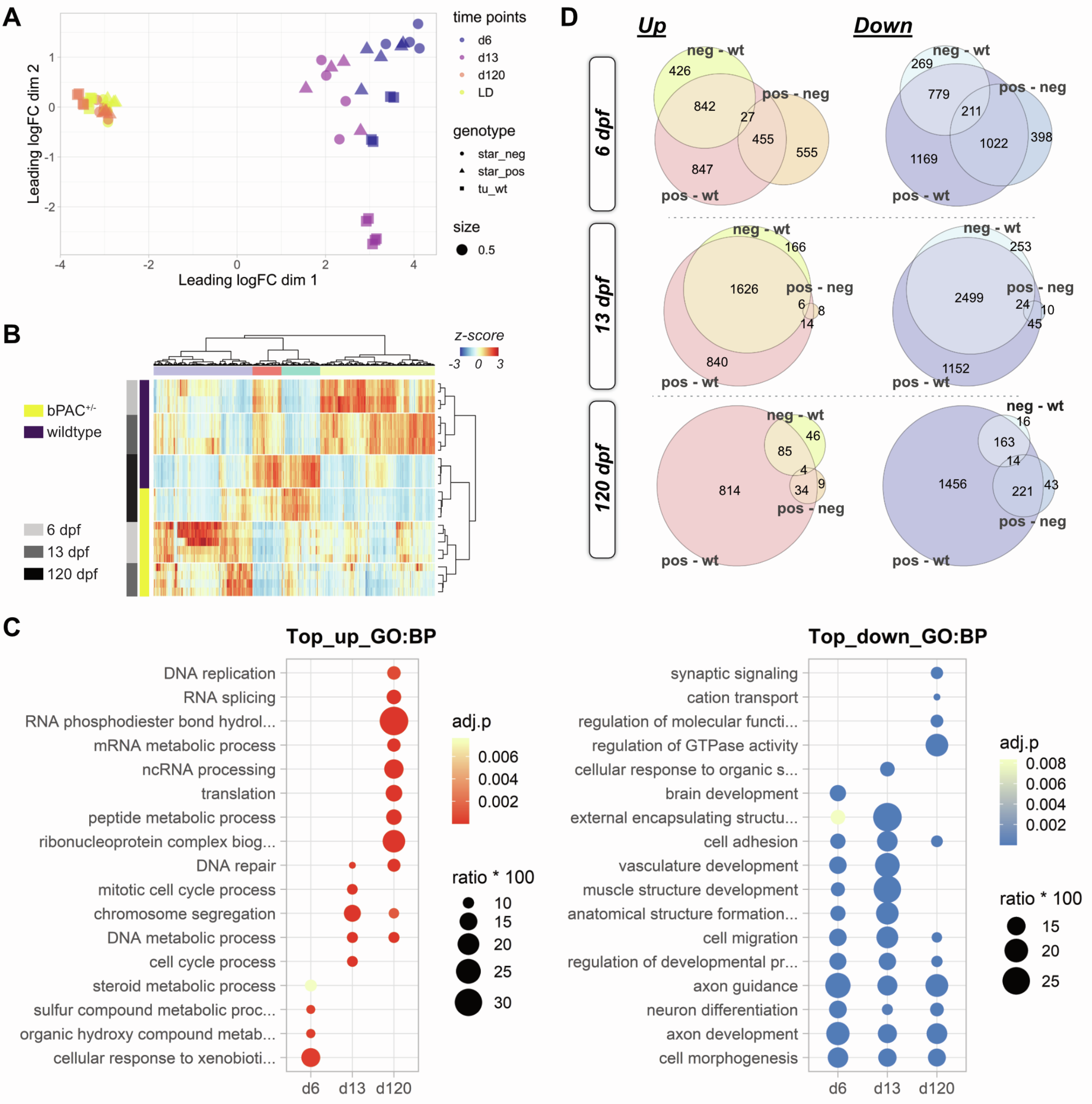

Figure S2. DEG identification and clustering (related to Figure 3). (A) MDS plot for RNA-seq data. Global profiles of samples were clustered by time and genotype. Profiles of bPAC+ and bPAC- were more similar to each other than to those of wild-type fish (wt). (B) Read counts of genes by genotype and time points are represented by z-score in the heat map using bPAC+ and wt samples. Bars on the left side indicate the genotype of fish and time points. (C) Top GO terms for biological processes enriched in upregulated (left) or downregulated (right) DEGs for the comparisons between bPAC+ and wt. d6: 6 dpf, d13: 13 dpf, d120: 120 dpf, wt: wild type, FC: fold change, GC: glucocorticoid, adj.p: adjusted P-value, ratio: identified DEGs/the number of genes included in the term. Complete DEG-enriched GO terms are described in Table S2. (D) Venn Diagram for up- and down-regulated DEGs among wt, bPAC- and bPAC+ by developmental stages.

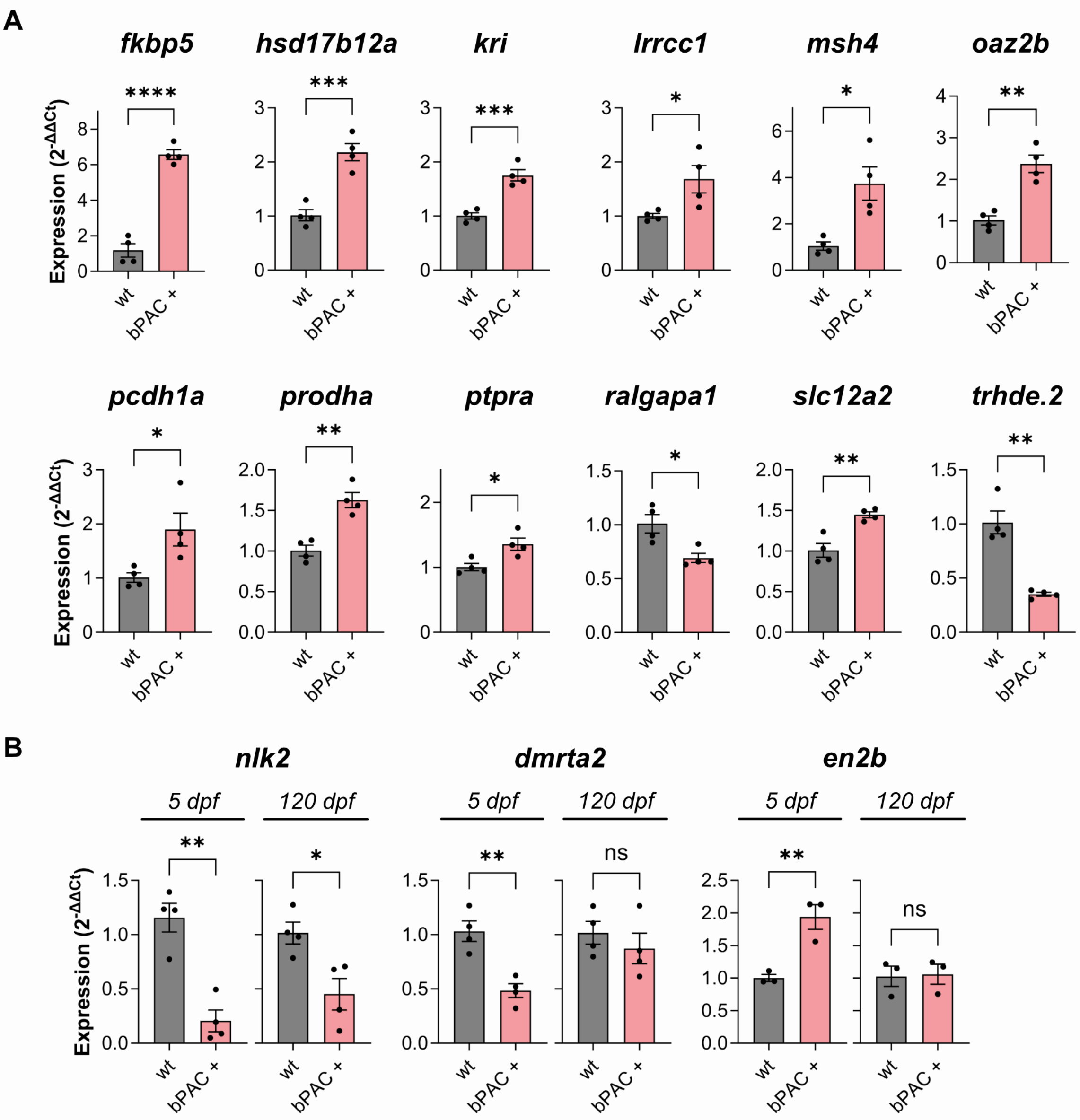

Figure S3. Gene expression validation of identified DEGs (related to Figure 3). Differential expression of a subset of identified DEGs was validated by real-time qPCR with independent biological replicates; (A) Whole body samples of wild types and bPAC+ at 5 dpf; (B) Brain samples of wild types and bPAC+ at 5 and 120 dpf. The majority of relative gene expressions among randomly selected DEGs conformed well to the trend of differential gene expression in RNA-seq results, except *en2b* expression in the 120 dpf brains. Expression of the gene was normalized by *18s* expression. *=P<0.05, **=P<0.01, ***=P<0.001, ****=P<0.0001. Two-tailed unpaired t-test. Error bar indicates mean±SEM.

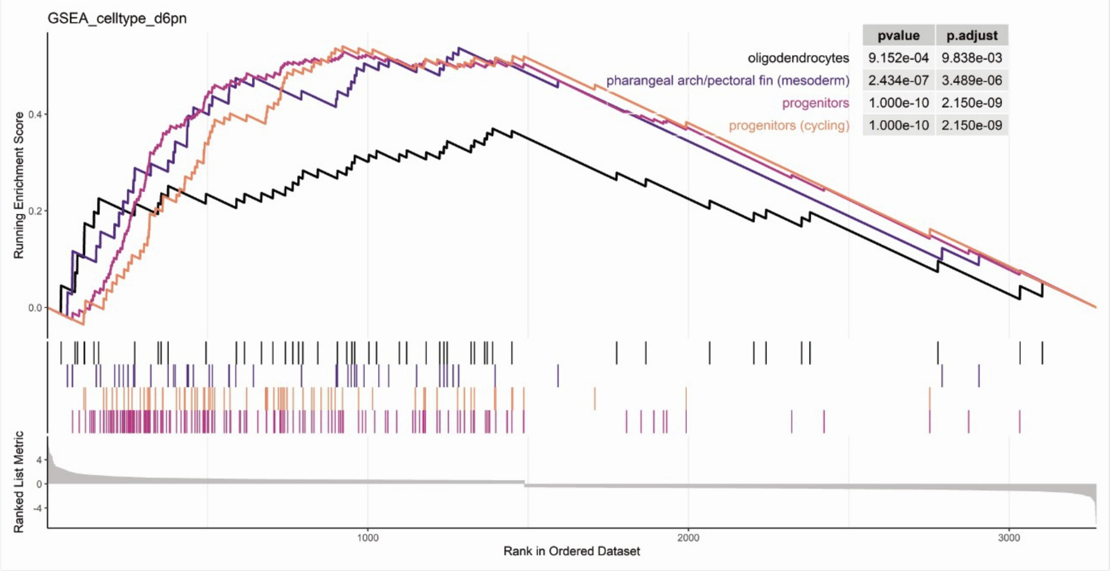

Description	setSize	enrichment Score	NES	qvalue	rank
progenitors	153	0.53	3.97	1.68E-09	923
progenitors(cycling)	72	0.54	3.30	1.68E-09	921
pharangealarch/pectoral fin (mesoderm)	45	0.54	2.83	2.73E-06	1283
oligodendrocytes	49	0.37	2.02	7.71E-03	1389

*NES: normalized enrichment score

Figure S4. Gene Set Enrichment Assay (GSEA) using 5 dpf whole-brain single-cell data (related to Figure 3). Top 250 markers for each cell cluster from the single-cell RNA-seq object (GSE158142_zf5dpf_cc_filt.cluster.rds) were used to create custom gene sets for all clusters for CNS clusters. The complete results of all GSEA analyses are available in Table S3.

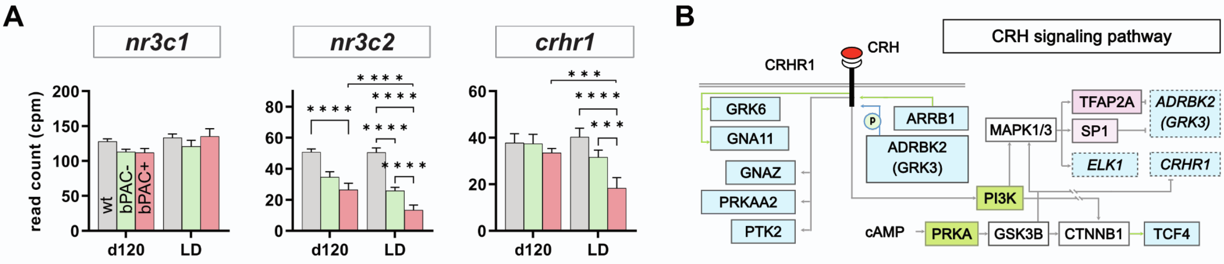

Figure S5. nr3c2 and crhr1 expressions were differentially regulated in bPAC+ following LD (related to Figure 4). (A Expression levels of nr3c1, nr3c2, and crhr1 before and after LD exposure. The asteria represents those comparisons that are statistically significant with |FC|>1.5. *=P<0.05, **=P<0.01, ***=P<0.001, ****=P<0.0001. Tukey's multiple comparisons test followed Restricted maximum likelihood analysis. Error bars indicate mean+SD. wt: wild type. (B) Following the LD exposure, several homologs in downstream of CRHR1 were differentially expressed in bPAC+ brains. Boxes colored light blue and pink indicate the downregulation and upregulation of its transcripts, respectively. The green-colored boxes represent enzyme complexes. The green and grey lines showed known and unknown interactions, respectively. The solid and dotted outlines of boxes represent protein and transcript, respectively. Pathway was sourced by WikiPathways (https://www.wikipathways.org/instance/WP2355).

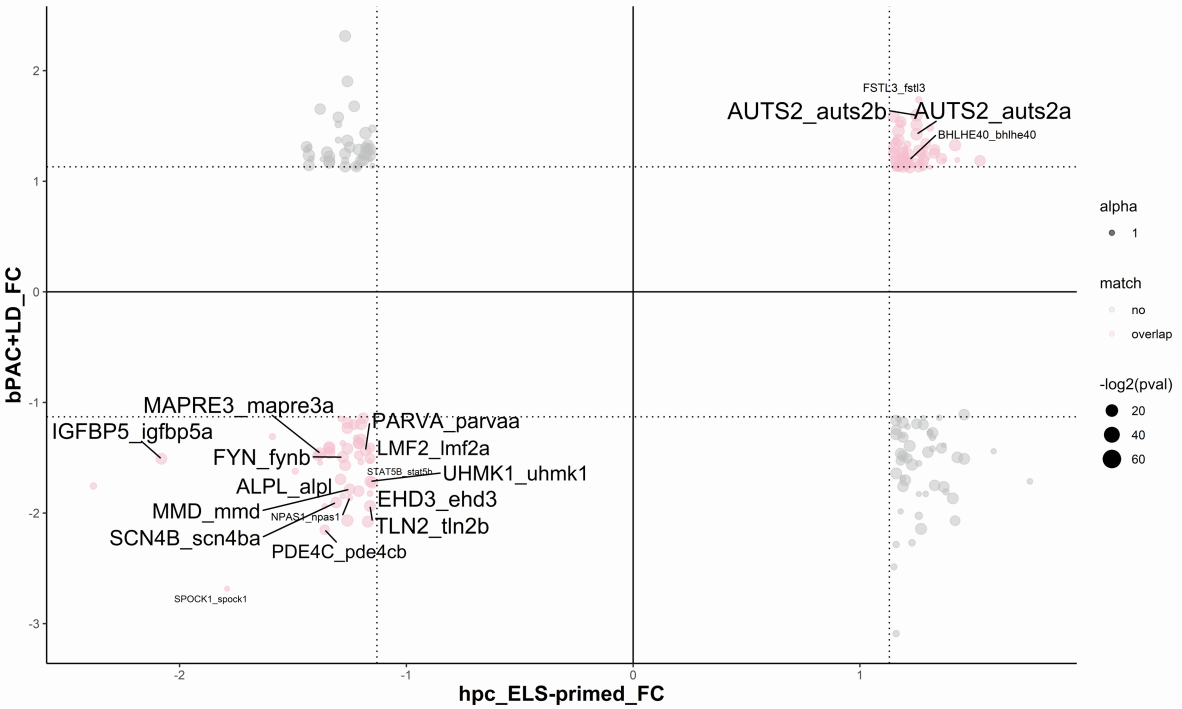

Figure S6. Comparison between bPAC+ post-LD genes and DEGs identified in the human hippocampal progenitor cell line (related to Figure 4). DEGs mapped to long-lasting differentially methylated cytosines from a GC-exposed human hippocampal progenitor cell line study (Provençal et al., 2020) were compared to bPAC+ post-LD DEGs (i.e. bPAC+/bPAC- post-LD comparison). X-axis represents the FC of post-LD DEGs in bPAC+, y-axis represents the FC of DEGs in GC-exposed mature neurons from a GC-exposed human hippocampal progenitor cell line. Labeled genes indicate GC-primed genes in both studies. Size of text for gene names indicates significance, -Log10(sqrt(P-values from studies)). Gene names are labeled as "human gene_zebrafish gene". Complete results are described in the Table S7.

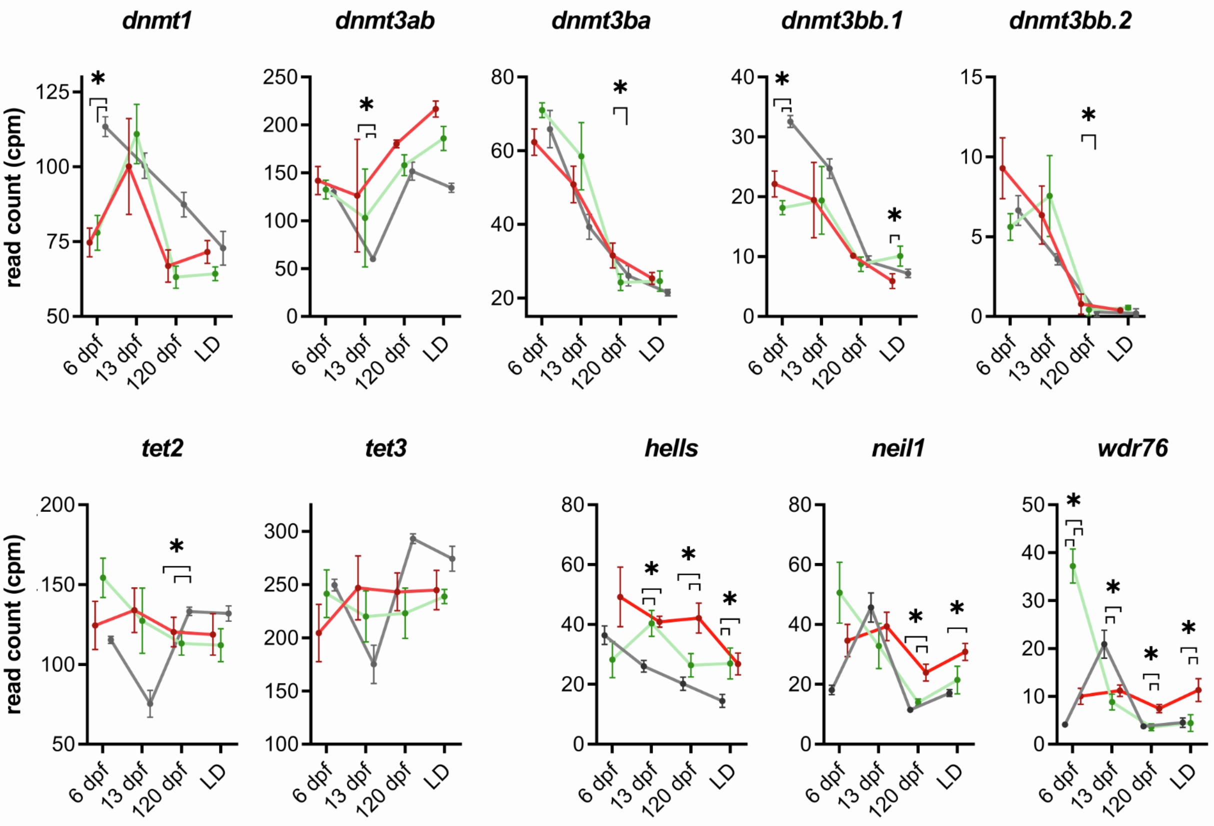

Figure S7. Expression trajectories of DNA methylation modulator genes (related to Figure 5A). 5mC writers (*dnmts*), 5mC erasers (*tets*), 5hmC readers (*hells*, *neil1*, *wdr76*). Red line: bPAC+, green line: bPAC-, grey line: wild type. The asteria mark indicates significant differential expression (FDR < 0.05, lFCl > 1.5).

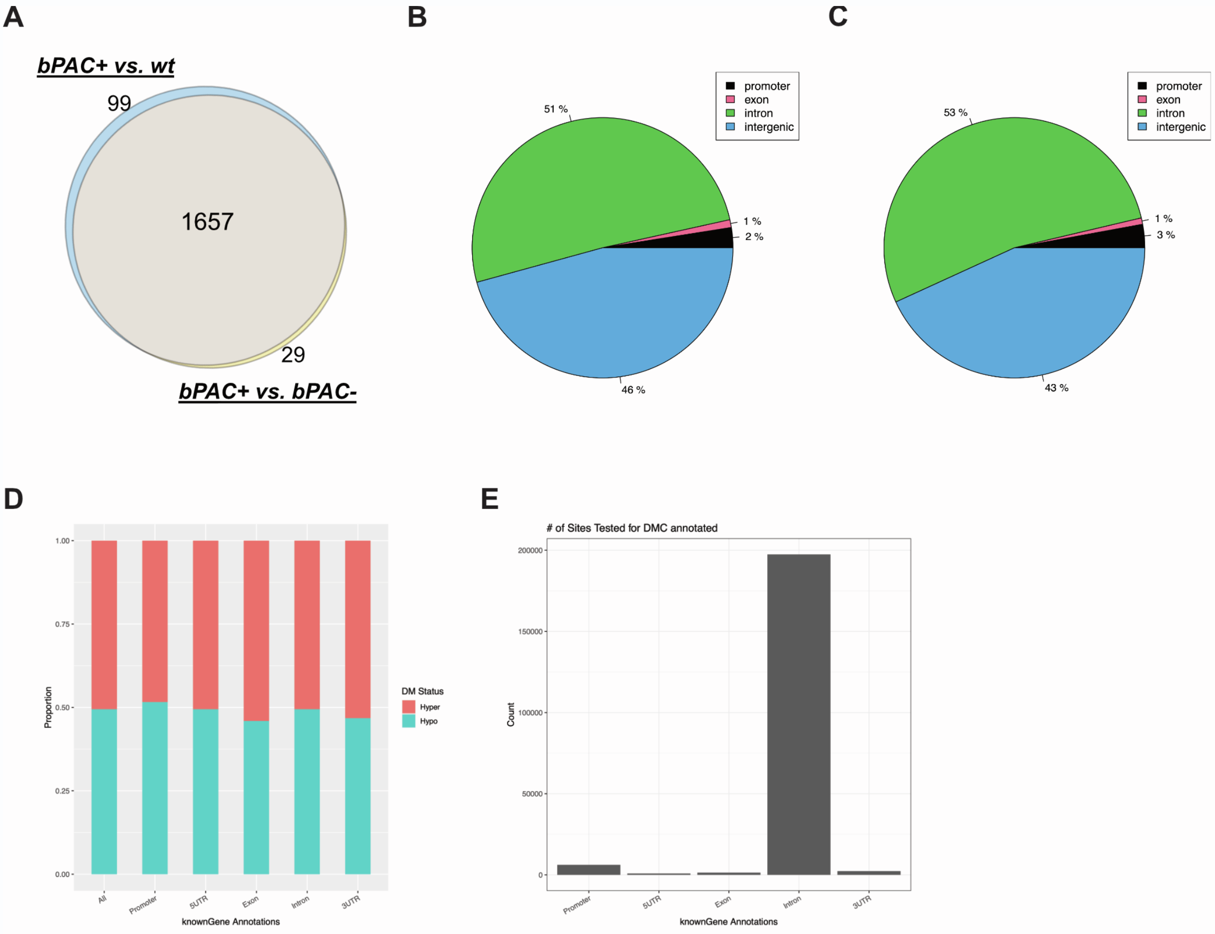

Figure S8. Identification of differentially methylated CpGs (DMCs) (related to Figure 5). (A) Venn diagram showing hdGC-primed genes from two pairwise comparisons, bPAC+ vs. wild type (wt) and bPAC+ vs. bPAC-. Distribution of Hypo- (B) and Hyper-methylated (C) DMCs according to the gene regions represented by Pie chart. (D) Proportion of Hypo and Hyper-methylated DMCs according to the gene regions represented by Bar charts. (E) The number of identified DMCs according to the gene regions.

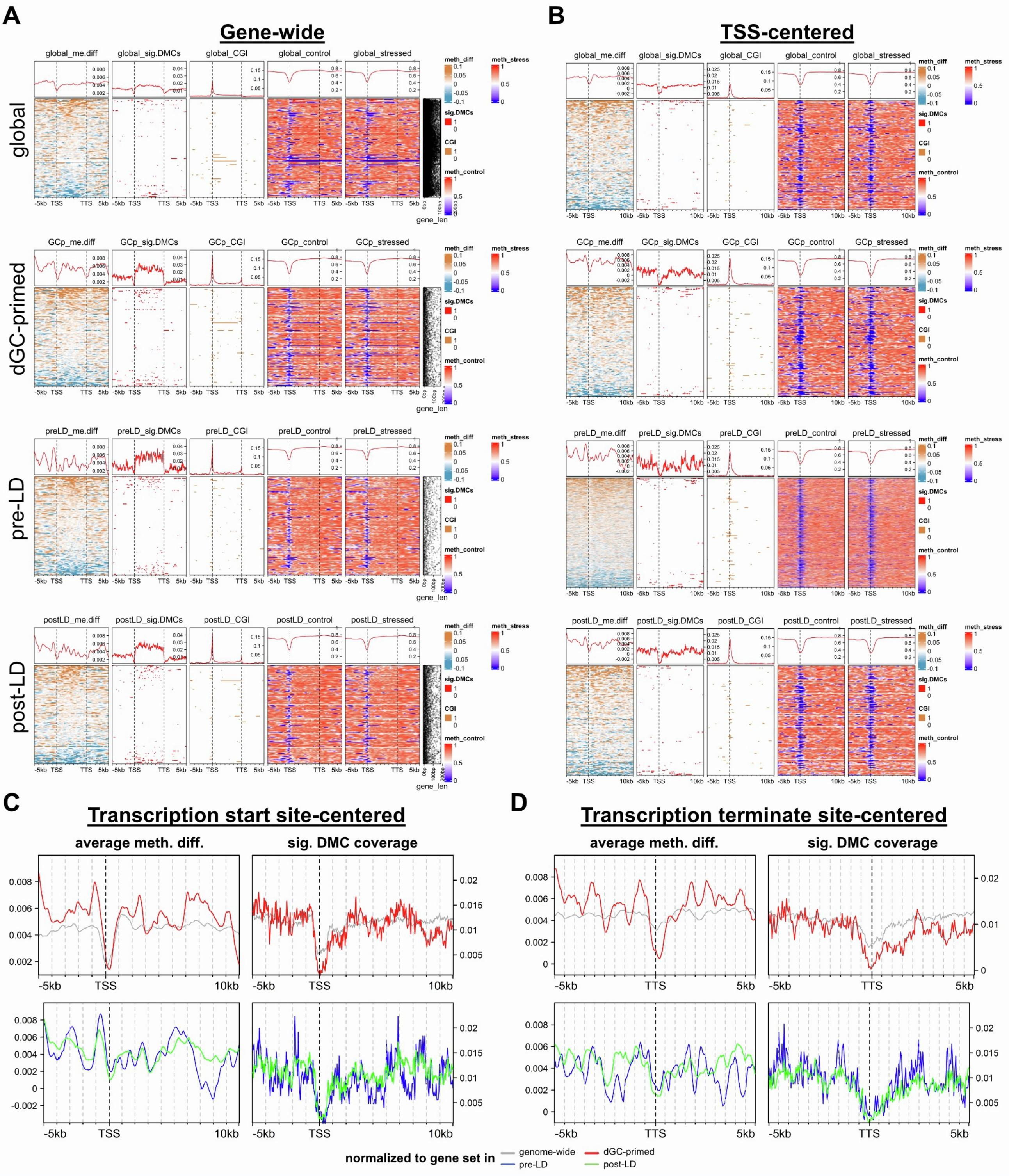

Figure S9. Coverage of methylated CpGs (DMCs) (related to Figure 5B). (A) The difference in mean methylation level of identified CpG sites, coverage of significant DMCs, CGI distribution, and mean methylation level of the control (wild type) and stressed (bPAC+) groups were visualized using a heatmap and line plot after normalization to the genic region. The mean methylation level difference (me_diff) was calculated as mean(wild type) - mean(bPAC+). (B) after normalization to the upstream 5kb and downstream 10kb region from the transcription start site (TSS). (C) Line plot after normalization to the upstream 5kb and downstream 10kb region from the TSS. (D) Line plot after normalization to the upstream 5kb and downstream 10kb region from the transcription termination site (TTS). TSS: transcription start site, TTS: transcription termination site, sig.:significant, meth_dif: methylation level difference.

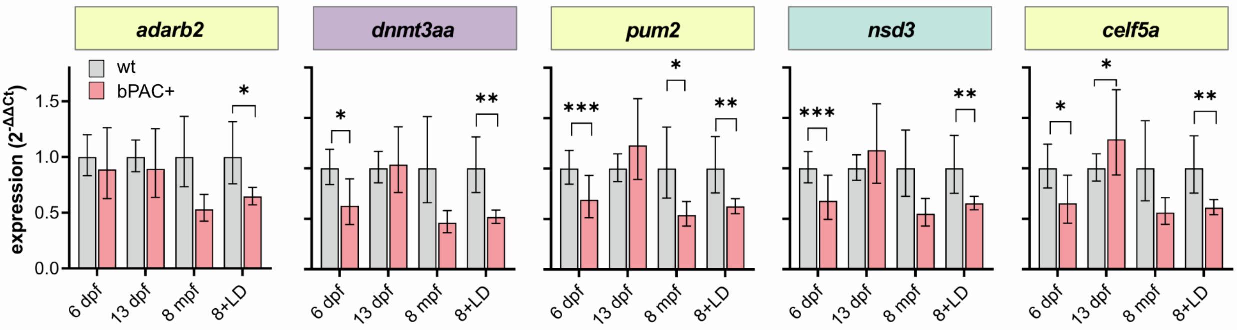

Figure S10. Validation of differential expression of identified GC-primed epigenetic modulator genes (related to Figure 5D-E). A subset of GC-primed genes in Figure 5G was selected and validated their expression by real-time qPCR with brain samples of both wild type and bPAC+ at 6, 13 dpf, and 8 mpf in basal and post-LD (+LD) conditions. The gray and pink bar represents wild type and bPAC+, respectively. *=P<0.05, **=P<0.01, ***=P<0.001, ****=P<0.0001, multiple unpaired t-test. dpf: day post-fertilization, mpf: month post-fertilization, wt: wild type.

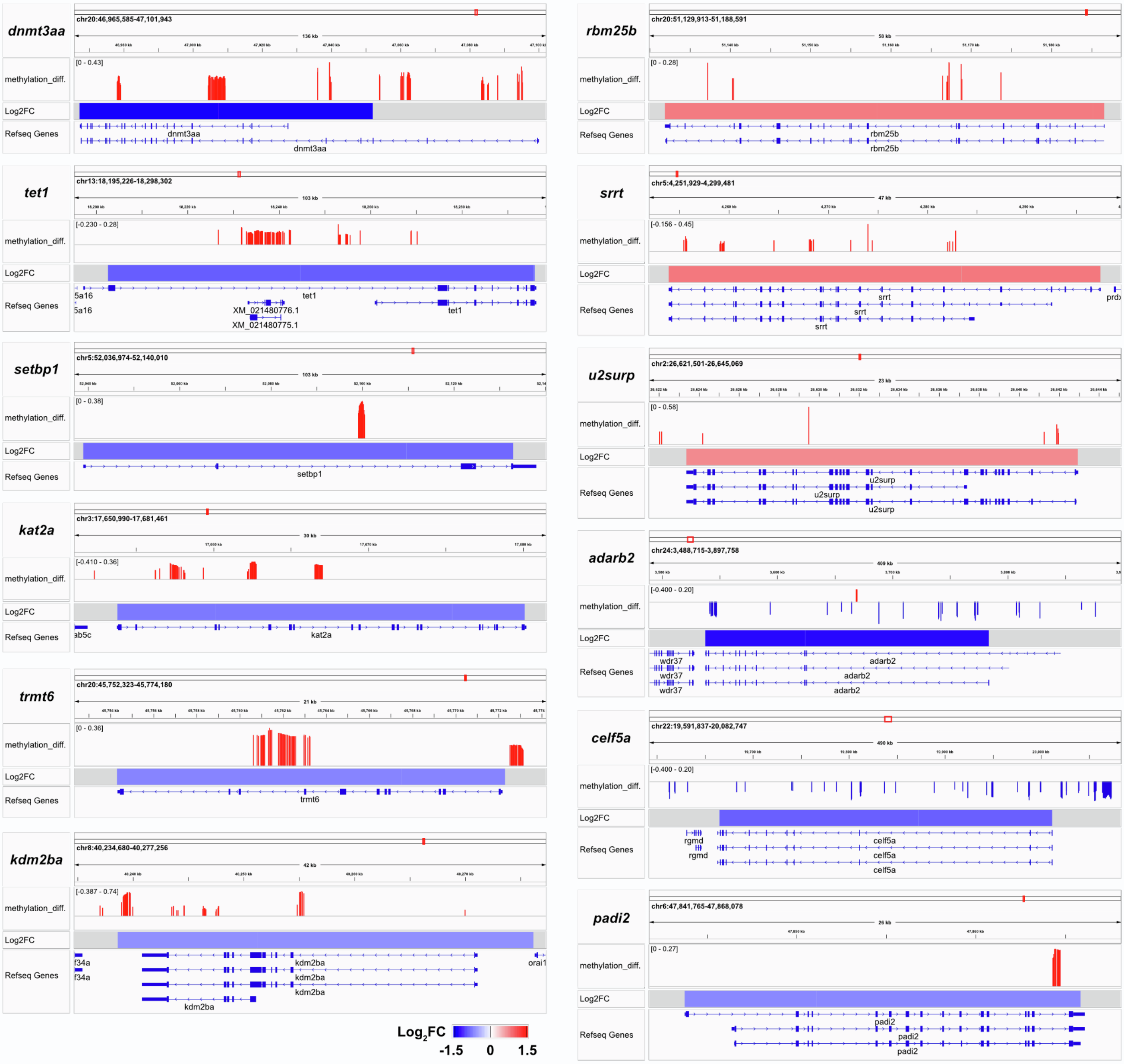

Figure S11. Distribution of significant DMCs on a subset of GC-primed epigenetic modulator genes (related to Figure 5F-G). Clusters of DMCs (at least 3 CpG in 100bp window) on 12 genes out of 48 GC-primed epigenetic modulator genes were identified and visualized by the IGV browser. methylation patterns of those genes were positively or negatively correlated with their gene expression pattern following LD-exposure. "methylation_diff" track represents differences in methylation level (-1 to 1, blue: -1 to 0, red: 0 to 1) of significant DMCs, which fit the criteria (difference in methylation level>25% and FDR<0.05). Gene expression level represented as Log2 fold change in "Log2FC" track. Gene annotation information was come from Refseq Genes (GRCZ11/danRer11).

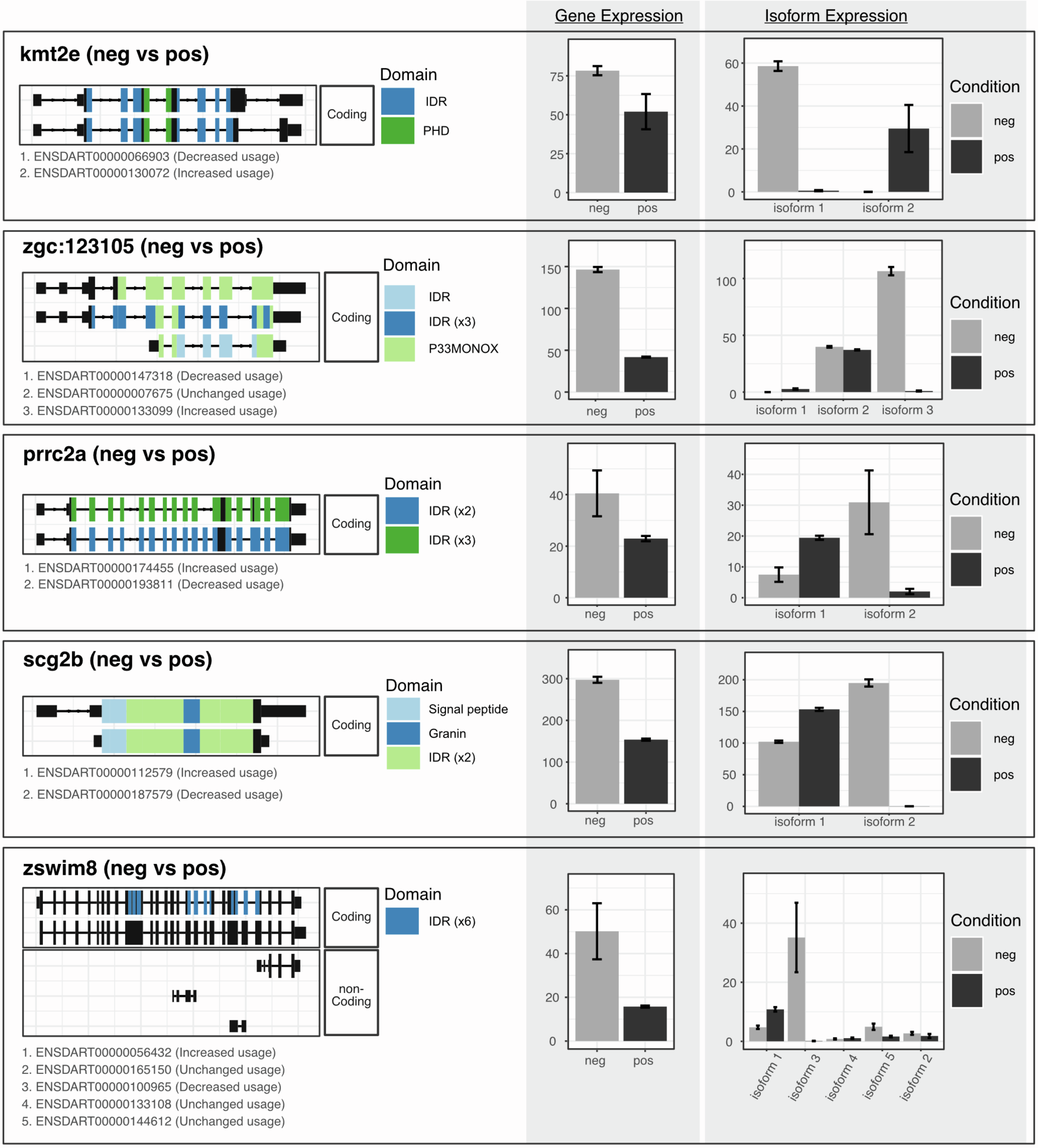

Figure S12. Significant isoform switches in brains following LD-exposure (related to Figure 5H). Significant isoform switches were selected by following the criteria: (1) Known transcript variants, (2) overall mean gene and isoform expression ≧ 5, (3) gene_switch_q_value < 0.01, and (4) dIF (differential isoform usage) > 0.4. The color code represents protein domains. Expression values are based on count per million (CPM). IDR: intrinsically disordered regions, PHD: plant homeodomain, P33MONOX: Putative monooxygenase p33MONOX, neg: bPAC-, pos: bPAC+.
