# Supplementary material for: Exposure to elevated glucocorticoid during development primes altered transcriptional responses to acute stress in adulthood
Source: iScience. 2024 May 31;27(7):110160. doi: 10.1016/j.isci.2024.110160 (PMC11233911; doi:10.1016/j.isci.2024.110160)
Supplement: Data S1. Volcano plots for all pairwise comparisons (|FC|>1.5 and FDR<0.05), related to Figure 3B [file mmc2.zip › Data S1. Volcano plots for all pairwise comparisons (related to Figure 3B).pdf]

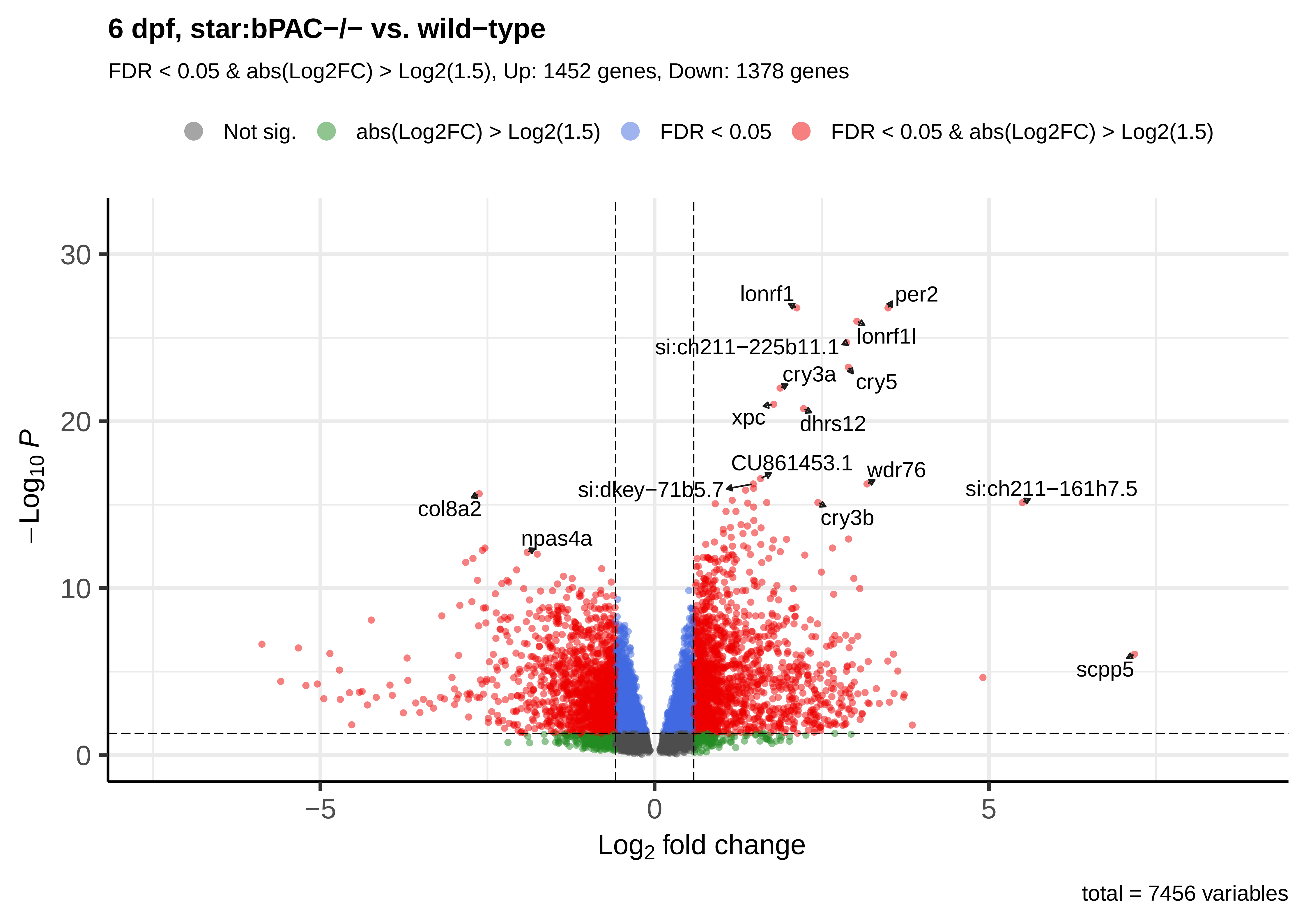
6 dpf, star:bPAC−/− vs. wild−type
FDR < 0.05 & abs(Log2FC) > Log2(1.5), Up: 1452 genes, Down: 1378 genes
Not sig.
abs(Log2FC) > Log2(1.5)
FDR < 0.05
FDR < 0.05 & abs(Log2FC) > Log2(1.5)
30
20
10
0
−5
0
5
−Log10 P
Log2 fold change
lonrf1
per2
lonrf1l
si:ch211−225b11.1
cry3a
cry5
xpc
dhrs12
CU861453.1
wdr76
si:dkey−71b5.7
si:ch211−161h7.5
col8a2
cry3b
npas4a
scpp5
total = 7456 variables

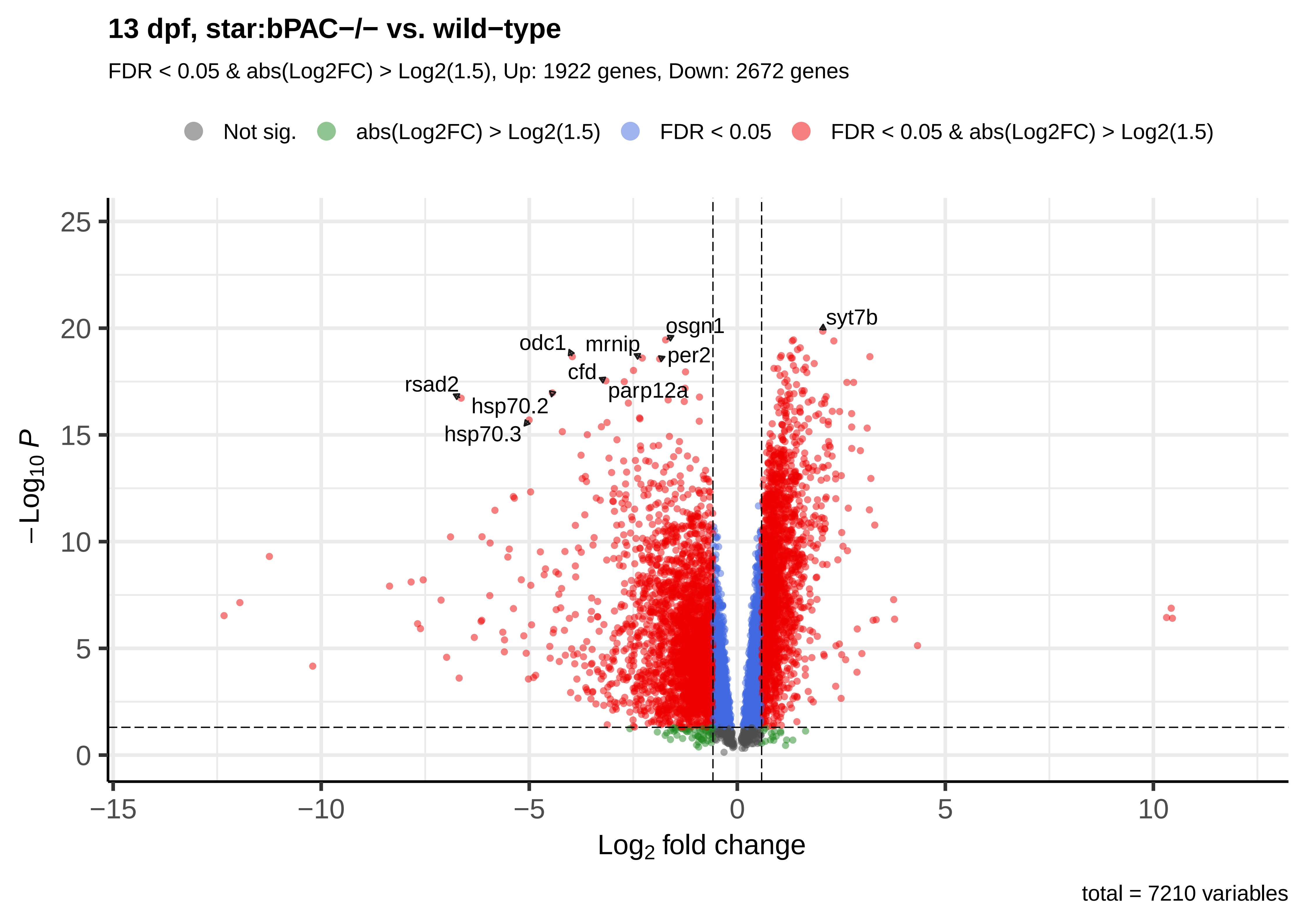

13 dpf, star:bPAC−/− vs. wild−type
FDR < 0.05 & abs(Log2FC) > Log2(1.5), Up: 1922 genes, Down: 2672 genes
Not sig.
abs(Log2FC) > Log2(1.5)
FDR < 0.05
FDR < 0.05 & abs(Log2FC) > Log2(1.5)
syt7b
osgn1
odc1
mrnip
per2
cfd
rsad2
parp12a
hsp70.2
hsp70.3
−Log10 P
Log2 fold change
total = 7210 variables

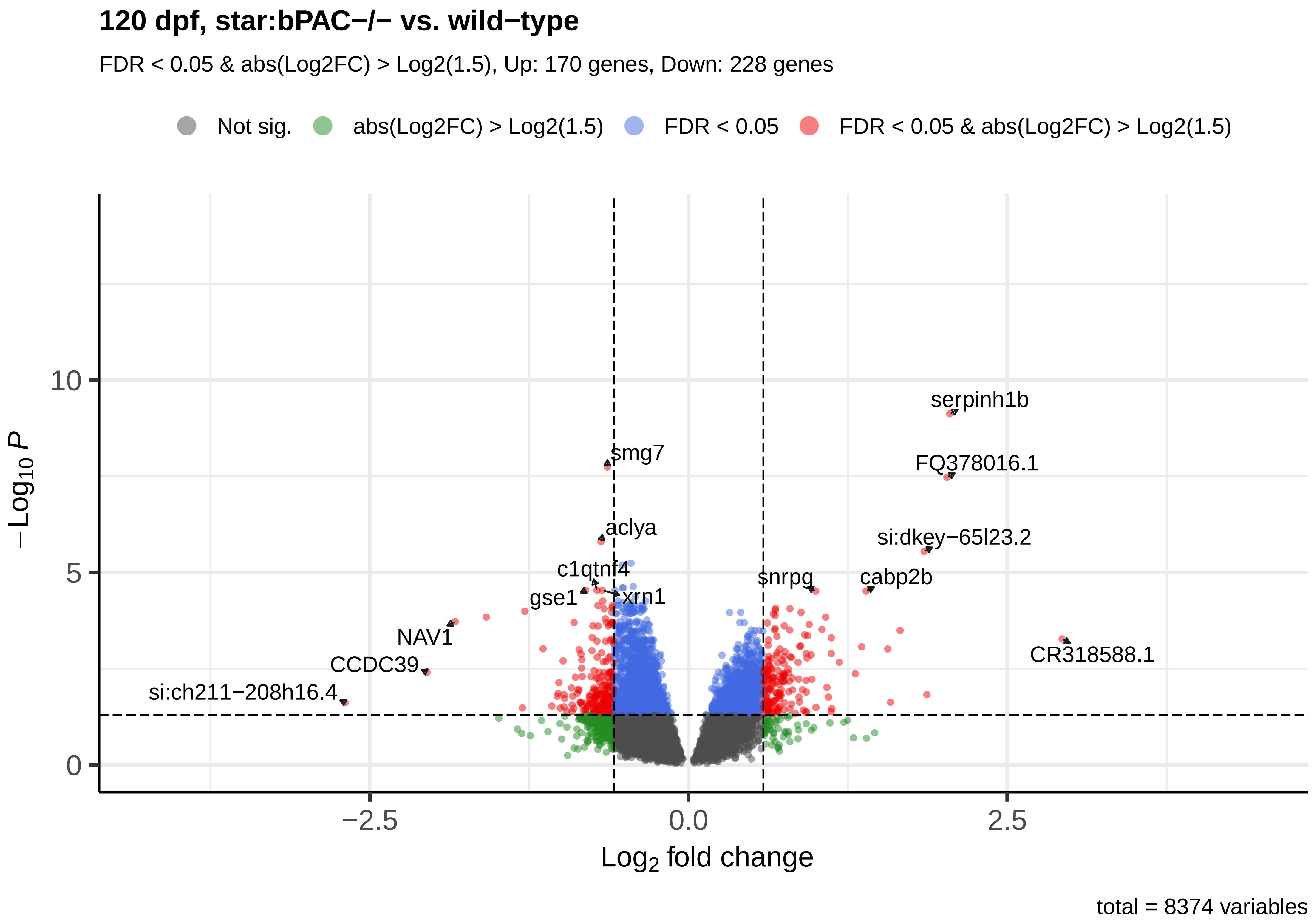
120 dpf, star:bPAC−/− vs. wild−type
FDR < 0.05 & abs(Log2FC) > Log2(1.5), Up: 170 genes, Down: 228 genes
Not sig.
abs(Log2FC) > Log2(1.5)
FDR < 0.05
FDR < 0.05 & abs(Log2FC) > Log2(1.5)
−Log10 P
10
5
0
serpinh1b
smg7
FQ378016.1
aclya
si:dkey−65l23.2
c1qtnf4
snrpg
cabp2b
gse1
xrn1
NAV1
CCDC39
CR318588.1
si:ch211−208h16.4
−2.5
0.0
2.5
Log2 fold change
total = 8374 variables

post−LD 120 dpf, star:bPAC−/− vs. wild−type

FDR < 0.05 & abs(Log2FC) > Log2(1.5), Up: 404 genes, Down: 1290 genes

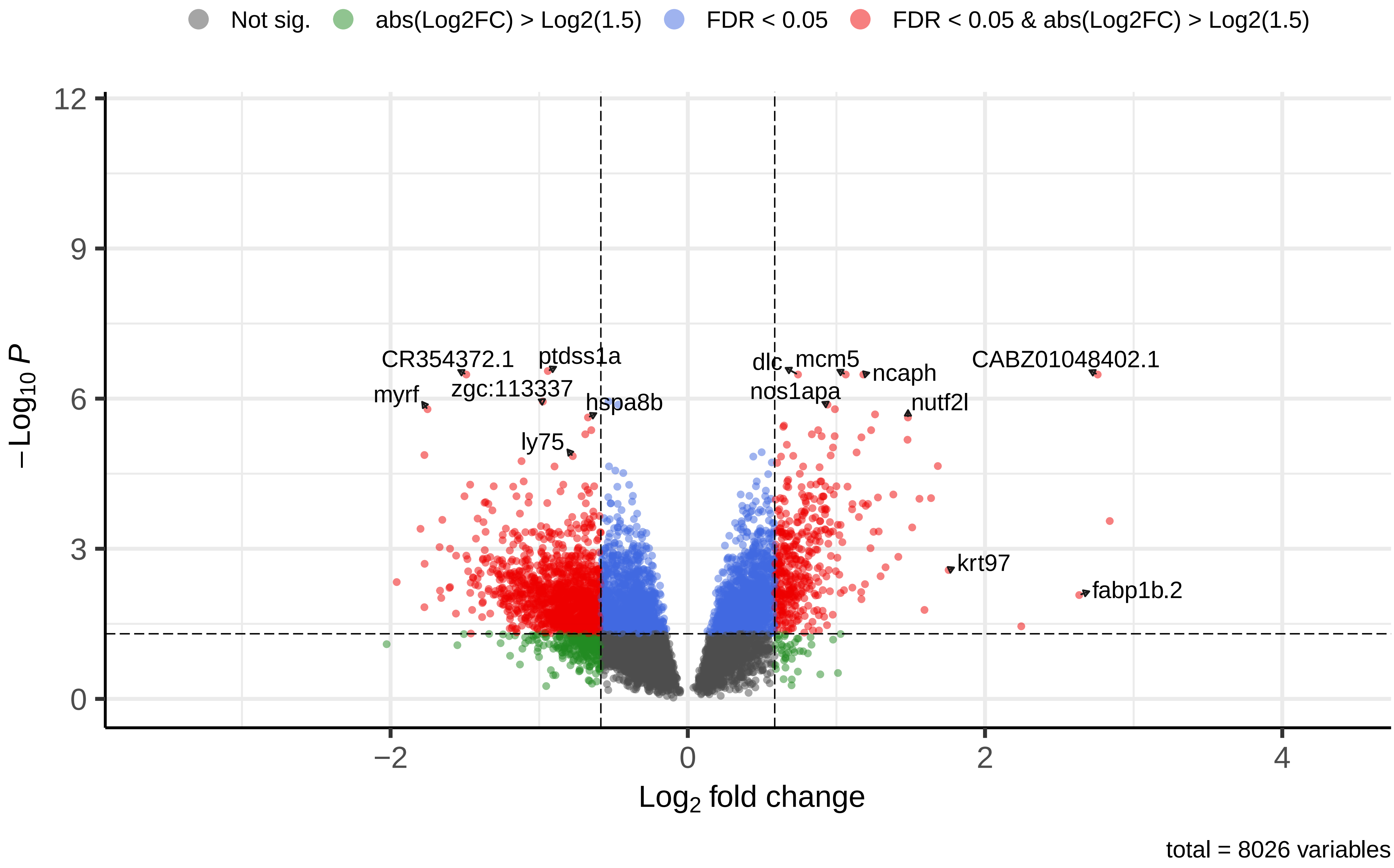

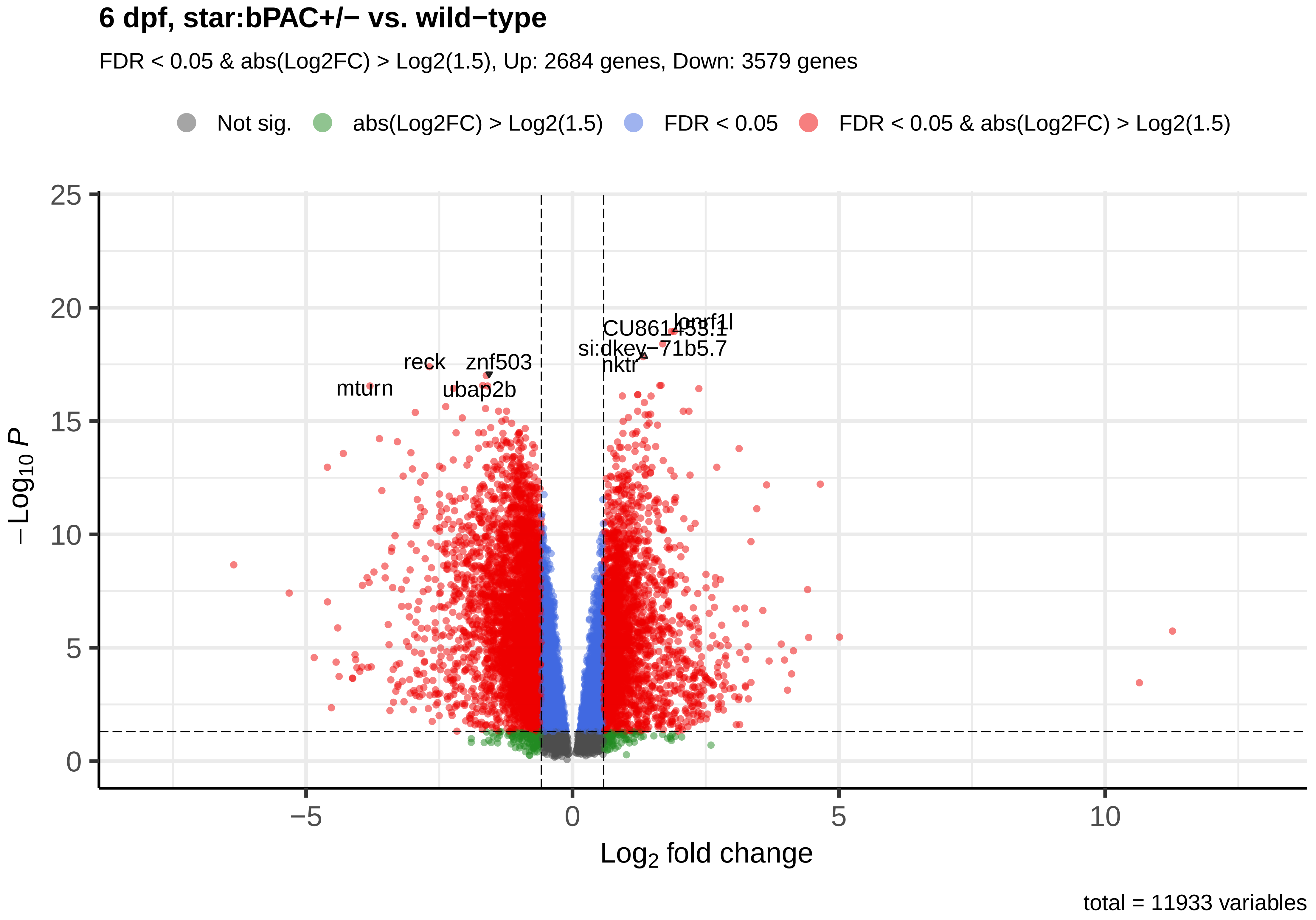
6 dpf, star:bPAC+/− vs. wild−type
FDR < 0.05 & abs(Log2FC) > Log2(1.5), Up: 2684 genes, Down: 3579 genes
Not sig.
abs(Log2FC) > Log2(1.5)
FDR < 0.05
FDR < 0.05 & abs(Log2FC) > Log2(1.5)
25
20
15
10
5
0
−Log10 P
mturn
reck
znf503
ubap2b
lonrf1l
CU861453.1
si:dkey−71b5.7
nktr
−5
0
5
10
Log2 fold change
total = 11933 variables

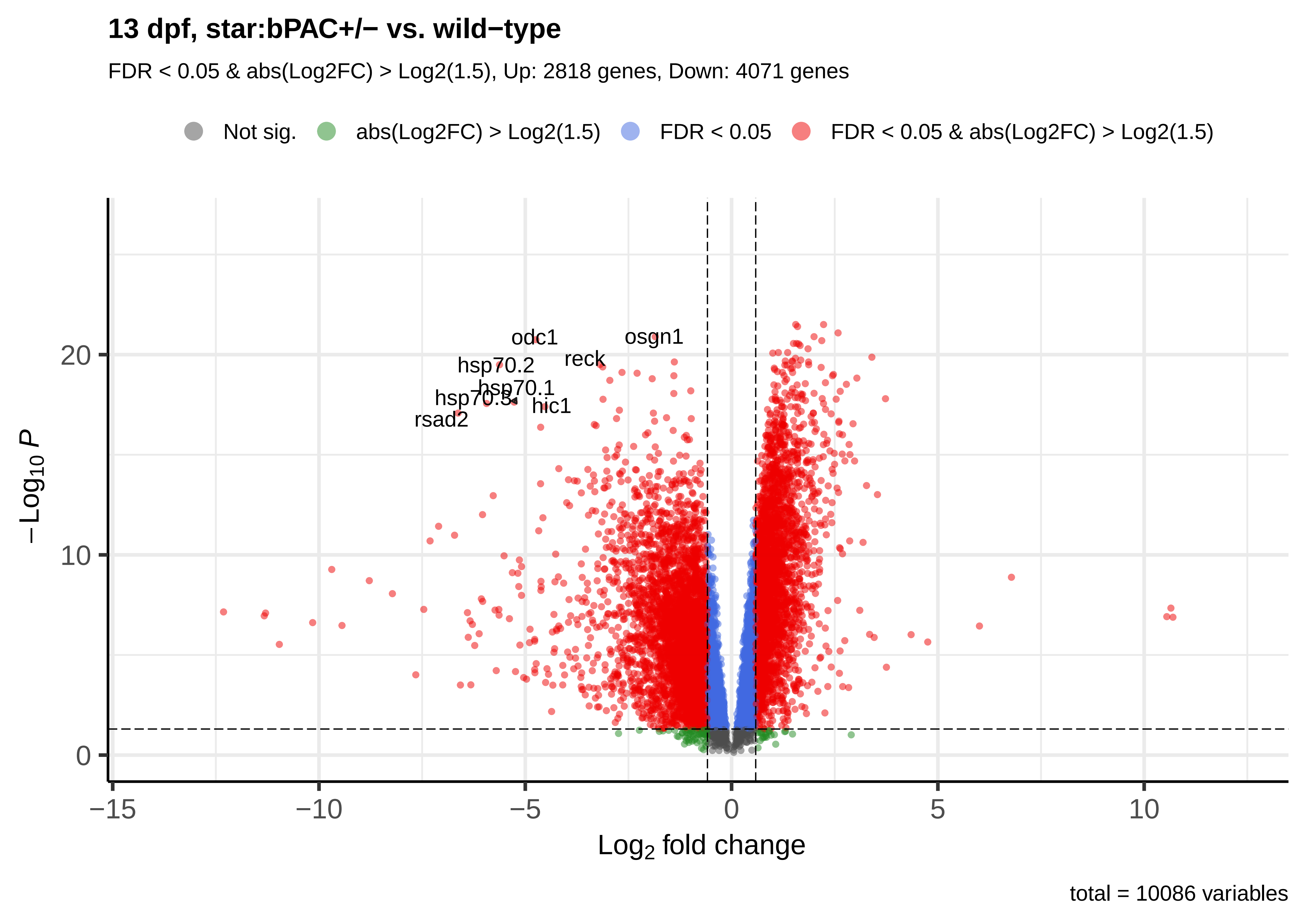
13 dpf, star:bPAC+/− vs. wild−type
FDR < 0.05 & abs(Log2FC) > Log2(1.5), Up: 2818 genes, Down: 4071 genes
Not sig.
abs(Log2FC) > Log2(1.5)
FDR < 0.05
FDR < 0.05 & abs(Log2FC) > Log2(1.5)
odc1
osgn1
reck
hsp70.2
hsp70.1
hsp70.3
hic1
rsad2
20
10
0
−15
−10
−5
0
5
10
$-Log_{10}$ P
Log_2 fold change
total = 10086 variables

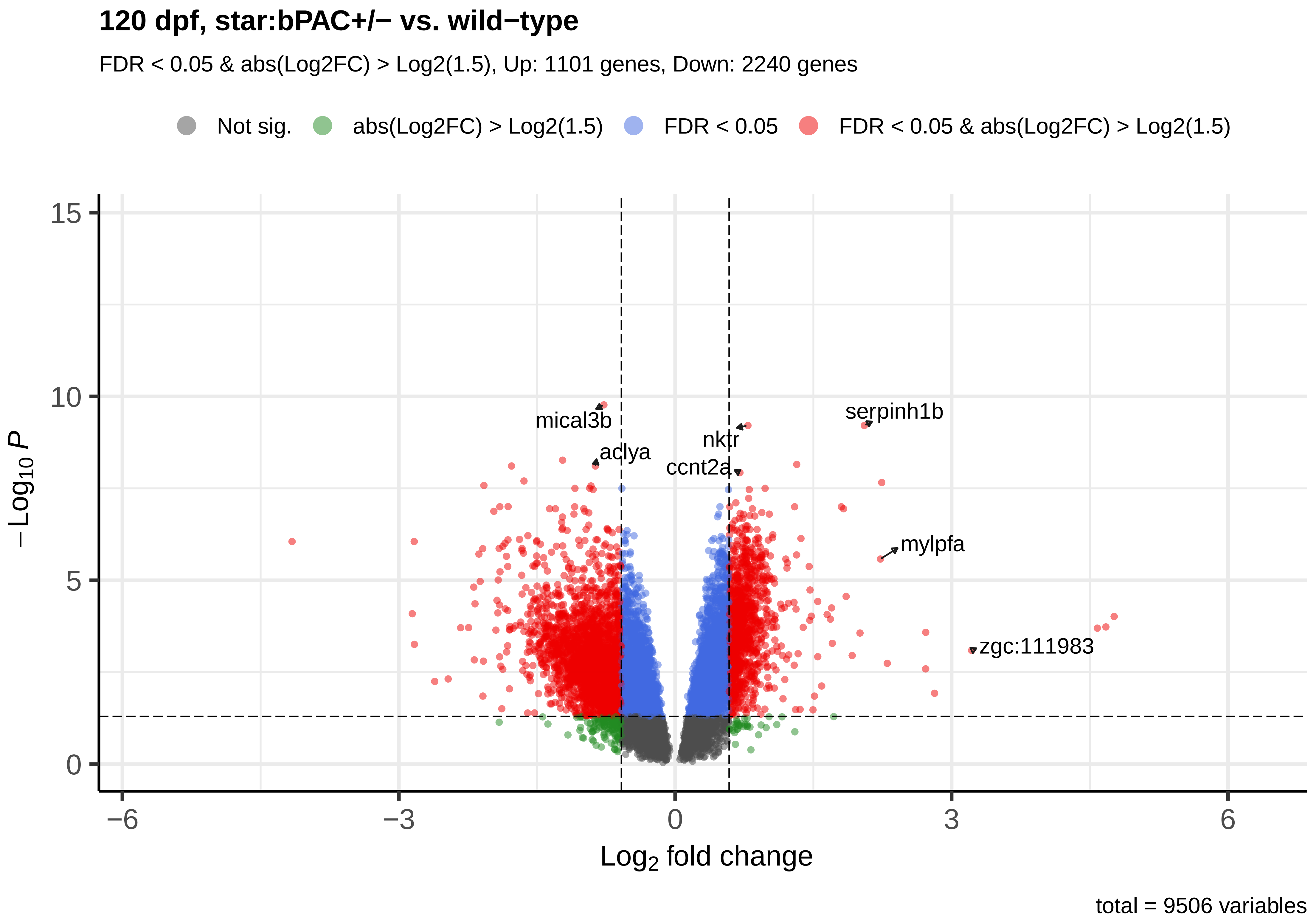
120 dpf, star:bPAC+/− vs. wild−type
FDR < 0.05 & abs(Log2FC) > Log2(1.5), Up: 1101 genes, Down: 2240 genes
Not sig.
abs(Log2FC) > Log2(1.5)
FDR < 0.05
FDR < 0.05 & abs(Log2FC) > Log2(1.5)
15
10
5
0
−6
−3
0
3
6
$-\text{Log}_{10}\,P$
Log_2 fold change
mical3b
aclya
nktr
ccnt2a
serpinh1b
mylpfa
zgc:111983
total = 9506 variables

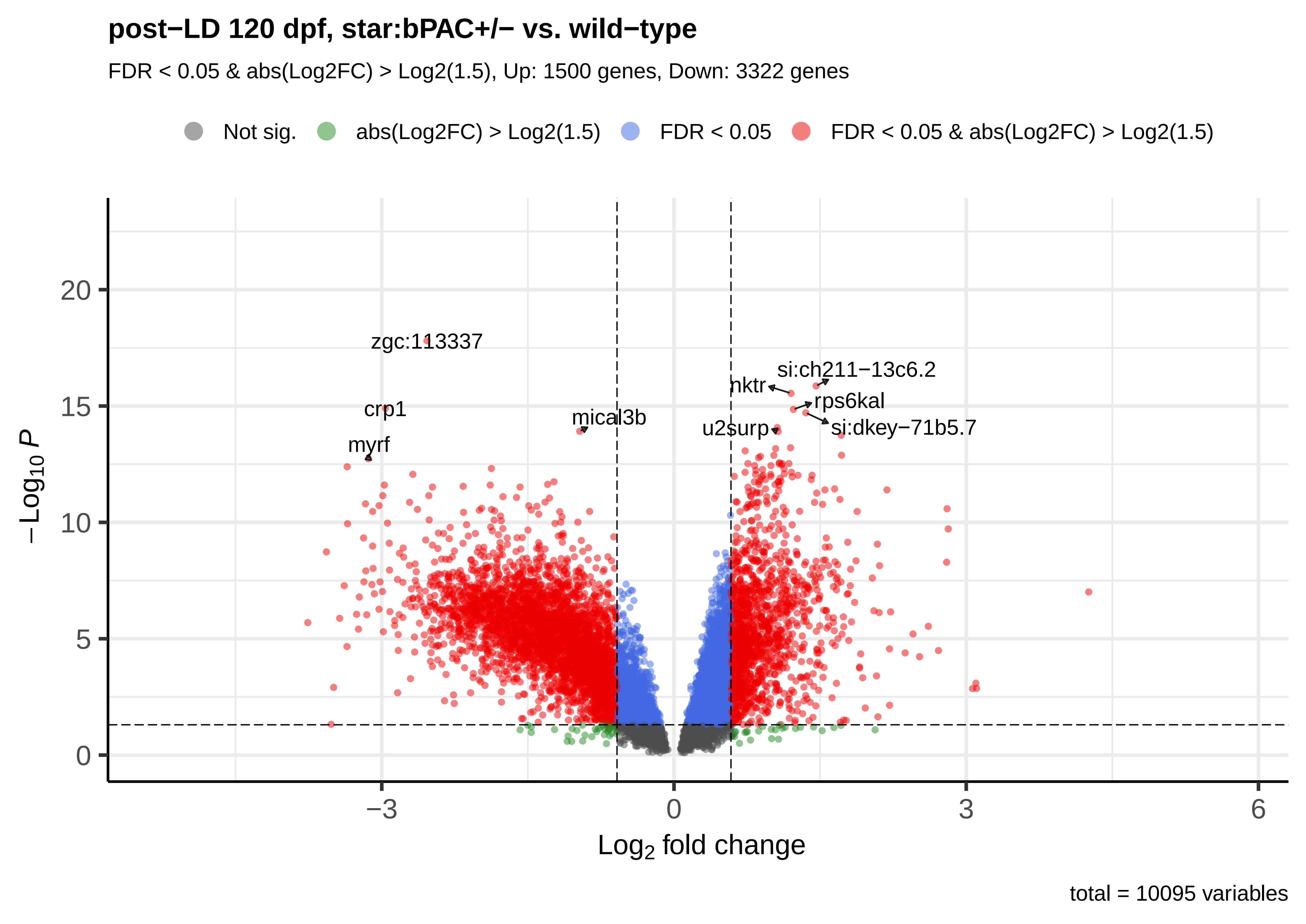

post−LD 120 dpf, star:bPAC+/− vs. wild−type
FDR < 0.05 & abs(Log2FC) > Log2(1.5), Up: 1500 genes, Down: 3322 genes
Not sig.
abs(Log2FC) > Log2(1.5)
FDR < 0.05
FDR < 0.05 & abs(Log2FC) > Log2(1.5)
zgc:113337
crp1
myrf
mical3b
nktr
si:ch211−13c6.2
rps6kal
u2surp
si:dkey−71b5.7
20
15
10
5
0
−3
0
3
6
$-\text{Log}_{10}\,P$
Log_2 fold change
total = 10095 variables

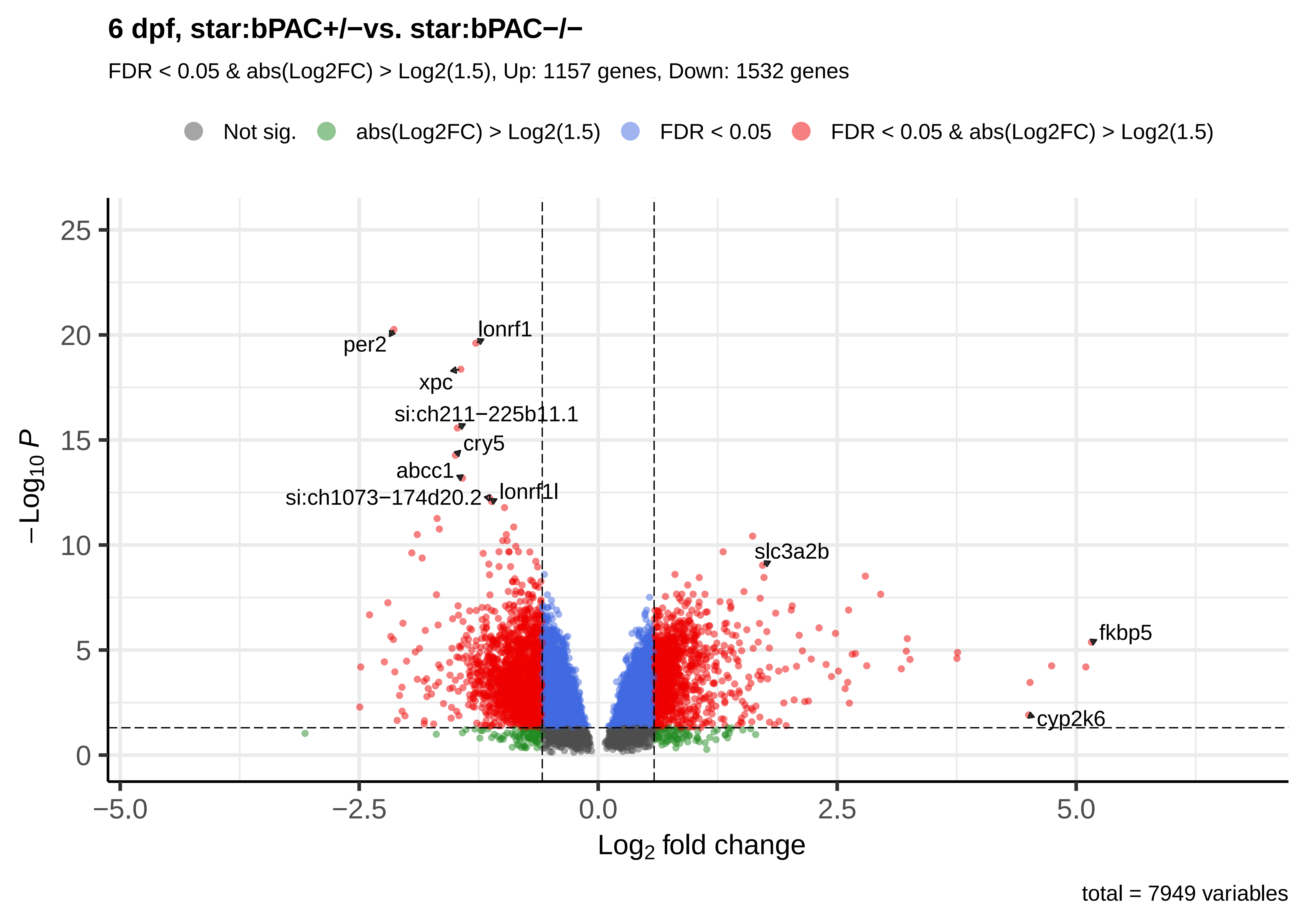
6 dpf, star:bPAC+/−vs. star:bPAC−/−
FDR < 0.05 & abs(Log2FC) > Log2(1.5), Up: 1157 genes, Down: 1532 genes
Not sig.
abs(Log2FC) > Log2(1.5)
FDR < 0.05
FDR < 0.05 & abs(Log2FC) > Log2(1.5)
per2
lonrf1
xpc
si:ch211−225b11.1
cry5
abcc1
si:ch1073−174d20.2
lonrf1l
slc3a2b
fkbp5
cyp2k6
25
20
15
10
5
0
−5.0
−2.5
0.0
2.5
5.0
$-\text{Log}_{10}\ P$
Log_2 fold change
total = 7949 variables

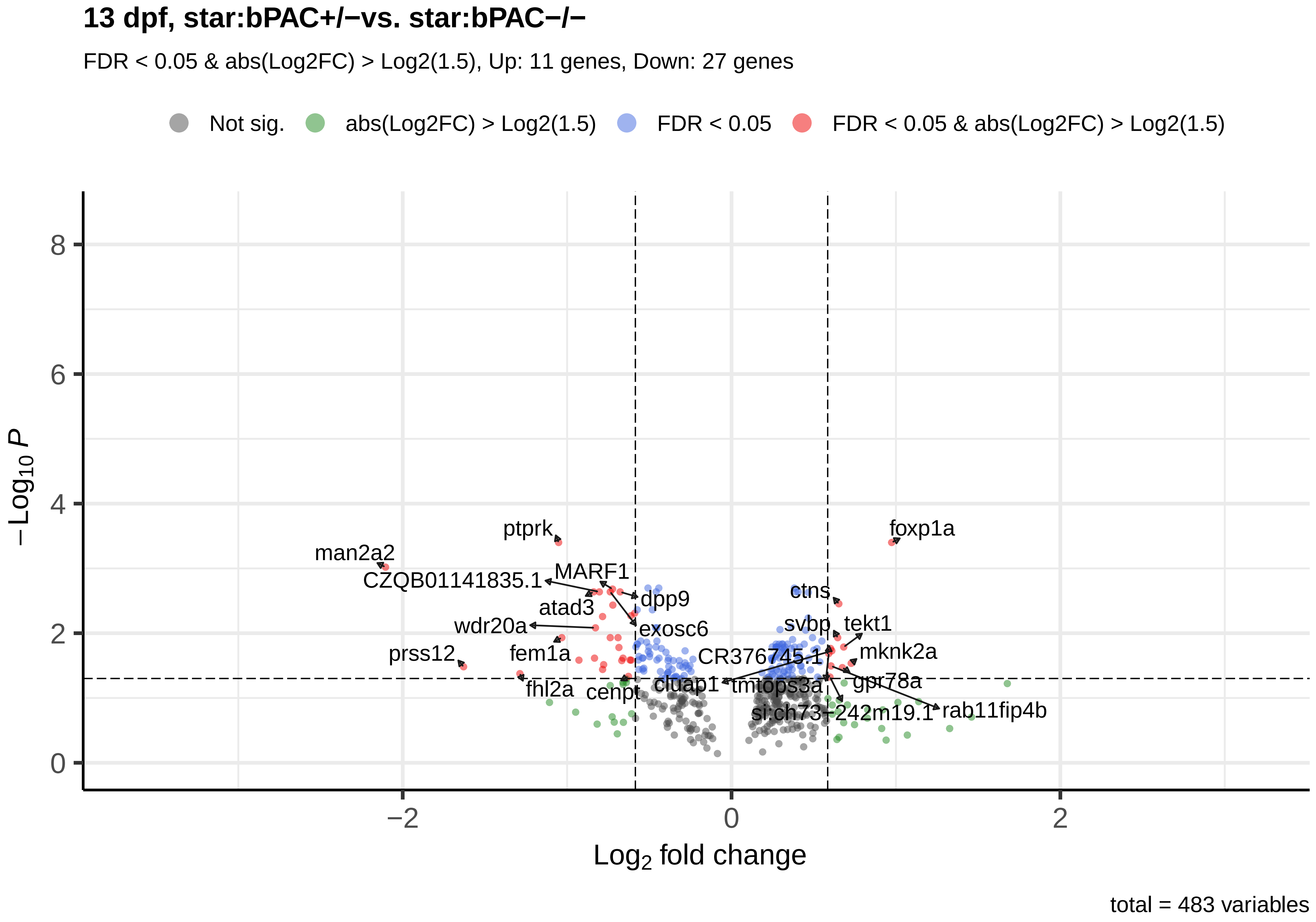

13 dpf, star:bPAC+/−vs. star:bPAC−/−
FDR < 0.05 & abs(Log2FC) > Log2(1.5), Up: 11 genes, Down: 27 genes
Not sig.
abs(Log2FC) > Log2(1.5)
FDR < 0.05
FDR < 0.05 & abs(Log2FC) > Log2(1.5)
−Log10 P
Log2 fold change
ptprk
man2a2
CZQB01141835.1
MARF1
atad3
dpp9
wdr20a
exosc6
prss12
fem1a
fhl2a
cenpt
cluap1
CR376745.1
tmtops3a
foxp1a
ctns
svbp
tekt1
mknk2a
gpr78a
rab11fip4b
si:ch73-242m19.1
total = 483 variables

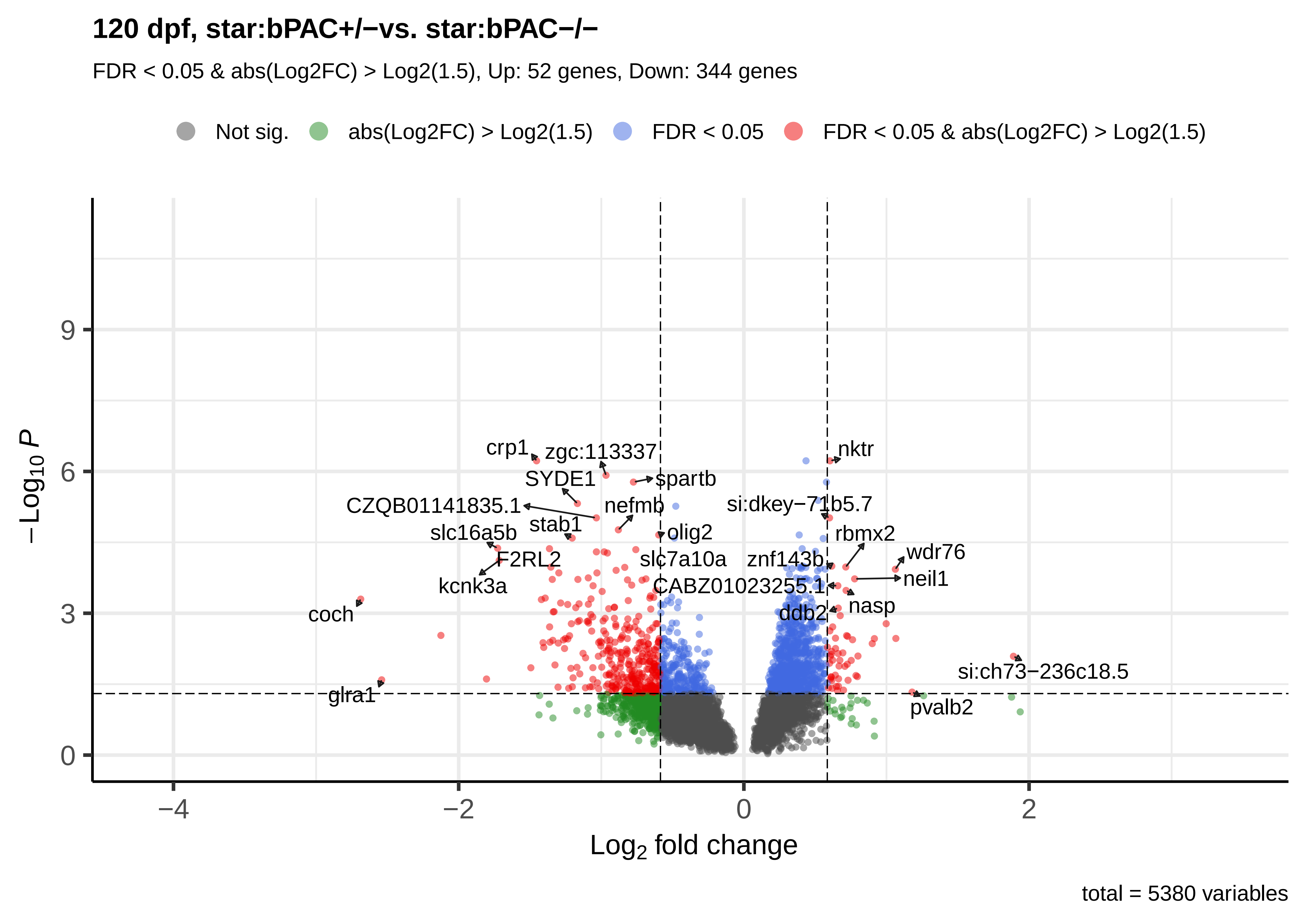
120 dpf, star:bPAC+/−vs. star:bPAC−/−
FDR < 0.05 & abs(Log2FC) > Log2(1.5), Up: 52 genes, Down: 344 genes
Not sig.
abs(Log2FC) > Log2(1.5)
FDR < 0.05
FDR < 0.05 & abs(Log2FC) > Log2(1.5)
crp1
zgc:113337
SYDE1
spartb
CZQB01141835.1
nefmb
stab1
slc16a5b
olig2
F2RL2
slc7a10a
kcnk3a
CABZ01023255.1
coch
glra1
nktr
si:dkey−71b5.7
rbmx2
znf143b
wdr76
neil1
nasp
ddb2
si:ch73−236c18.5
pvalb2
−Log10 P
Log2 fold change
9
6
3
0
−4
−2
0
2
total = 5380 variables

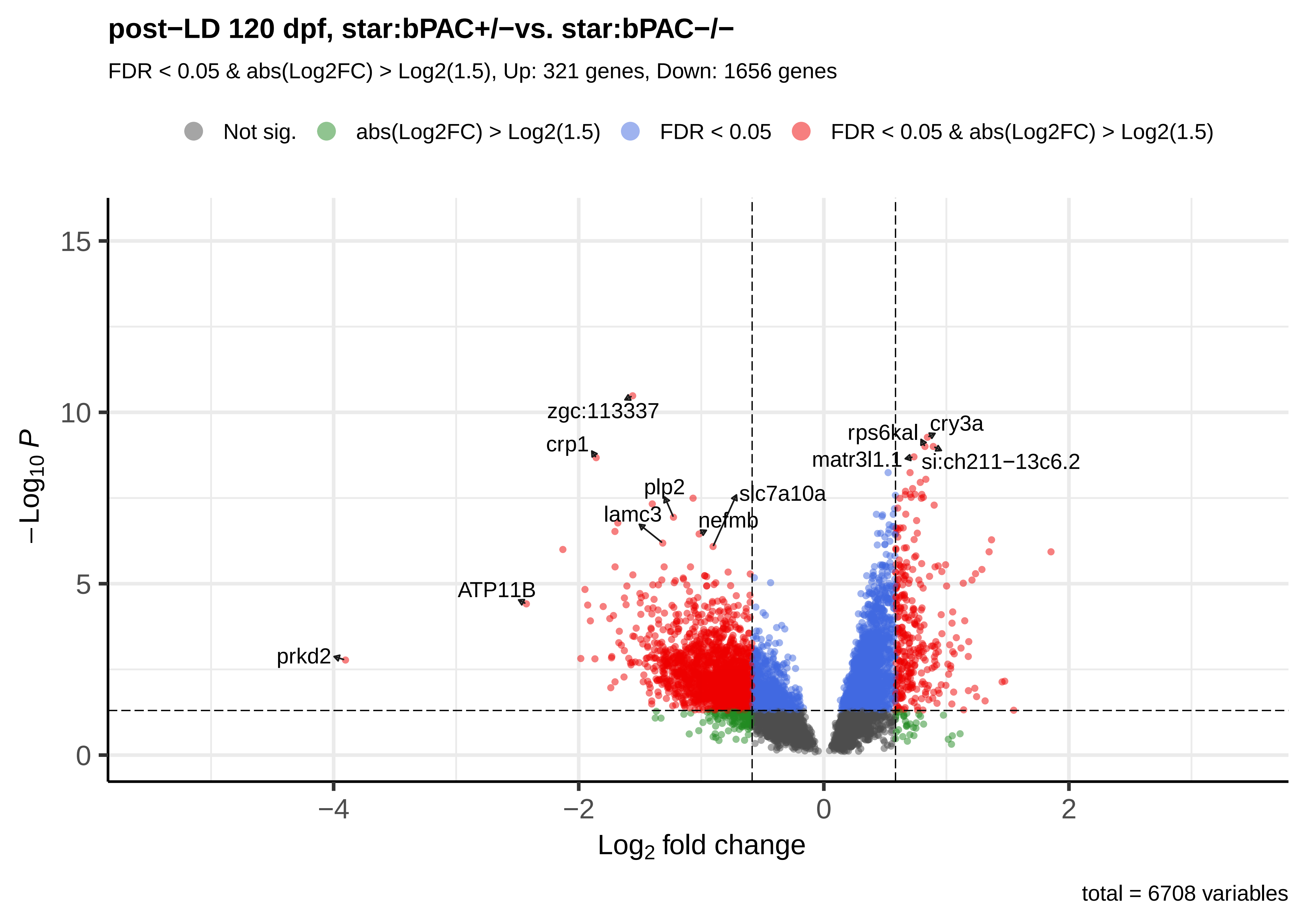
post−LD 120 dpf, star:bPAC+/−vs. star:bPAC−/−
FDR < 0.05 & abs(Log2FC) > Log2(1.5), Up: 321 genes, Down: 1656 genes
Not sig.
abs(Log2FC) > Log2(1.5)
FDR < 0.05
FDR < 0.05 & abs(Log2FC) > Log2(1.5)
zgc:113337
crp1
plp2
lamc3
slc7a10a
nefmb
ATP11B
prkd2
rps6kal
cry3a
matr3l1.1
si:ch211−13c6.2
15
10
5
0
−4
−2
0
2
$-\mathrm{Log}_{10}\,P$
Log_2 fold change
total = 6708 variables
